# Seven new microendemic species of *Brachycephalus* (Anura: Brachycephalidae) from southern Brazil

**DOI:** 10.7717/peerj.1011

**Published:** 2015-06-04

**Authors:** Luiz F. Ribeiro, Marcos R. Bornschein, Ricardo Belmonte-Lopes, Carina R. Firkowski, Sergio A.A. Morato, Marcio R. Pie

**Affiliations:** 1 Faculdade Dom Bosco, Curitiba Paraná, Brazil; 2 Mater Natura—Instituto de Estudos Ambientais, Curitiba Paraná, Brazil; 3 Departamento de Zoologia, Universidade Federal do Paraná, Curitiba Paraná, Brazil; 4 Programa de Pós-Graduação em Ecologia, Conservação e Manejo da Vida Silvestre, Universidade Federal de Minas Gerais, Belo Horizonte Minas Gerais, Brazil; 5 STCP Engenharia de Projetos Ltda, Curitiba Paraná, Brazil; 6 MBA Internacional em Gestão Ambiental, Universidade Federal do Paraná, Curitiba Paraná, Brazil

**Keywords:** Atlantic rainforest, Saddle-back toads, Cloud forest, Terrarana

## Abstract

*Brachycephalus* (Anura: Brachycephalidae) is a remarkable genus of miniaturized frogs of the Brazilian Atlantic Rainforest. Many of its species are highly endemic to cloud forests, being found only on one or a few mountaintops. Such level of microendemism might be caused by their climatic tolerance to a narrow set of environmental conditions found only in montane regions. This restriction severely limits the chance of discovery of new species, given the difficulty of exploring these inaccessible habitats. Following extensive fieldwork in montane areas of the southern portion of the Atlantic Rainforest, in this study we describe seven new species of *Brachycephalus* from the states of Paraná and Santa Catarina, southern Brazil. These species can be distinguished from one another based on coloration and the level of rugosity of the skin in different parts of their body. These discoveries increase considerably the number of described species of *Brachycephalus* in southern Brazil.

## Introduction

*Brachycephalus* Fitzinger, 1826 (Anura: Brachycephalidae) is a fascinating frog genus endemic to the Brazilian Atlantic Rainforest, known for its minute size and strong endemism, particularly in cloud forests (Pombal, Wistuba & Bornschein, 1998; Ribeiro et al., 2005; Alves et al., 2006; Napoli et al., 2011; Pie et al., 2013). The high level of microendemism found in some *Brachycephalus* species might be caused by their climatic tolerance to a narrow set of environmental conditions found only in montane regions (Pie et al., 2013). Such small geographical ranges limit the chance of discovery of new species, given the difficulty in exploring these inaccessible habitats. Several *Brachycephalus* species display conspicuous (aposematic) coloration patterns that are associated with the presence of tetrodotoxin and its analogues, which are highly potent neurotoxins that are particularly concentrated in their integument (Pires et al., 2002). Little is known about the natural history of *Brachycephalus*, and most of what is known about them is included in their original descriptions (but see Pombal, Sazima & Haddad, 1994; Van Sluys et al., 2007; Verdade et al., 2008; Almeida-Santos et al., 2011; Fontoura, Ribeiro & Pie, 2011; Dorigo et al., 2012; Rocha et al., 2013; Siqueira et al., 2014.

Several lines of evidence indicate the existence of three main groups within *Brachycephalus*, which currently includes 21 nominal species. The *ephippium* group (Spix, 1824) (Pie et al., 2013—clade 1 *sensu* Clemente-Carvalho et al., 2011) is present in southeastern Brazil (states of Rio de Janeiro, São Paulo and Espírito Santo) and includes most of the species of the genus known to date (*B. alipioi* Pombal & Gasparini, 2006, *B. ephippium* (Spix, 1824), *B. garbeana* Miranda-Ribeiro, 1920, *B. nodoterga* Miranda-Ribeiro, 1920, *B. pitanga* Alves, Sawaya, Reis & Haddad, 2009, *B. toby* Haddad, Clemente-Carvalho, and Reis, 2010, and *B. vertebralis* Pombal & Izecksohn 2001). The *pernix* group Pombal, Wistuba & Bornschein, 1998 (Pie et al., 2013—clade 2 *sensu* Clemente-Carvalho et al., 2011) includes the species present in southern Brazil (state of Paraná), namely *B. pernix* Pombal, Wistuba & Bornschein, 1998, *B. brunneus* Ribeiro et al., 2005, *B. izecksohni* Ribeiro et al., 2005, *B. ferruginus* Alves et al., 2006, and *B. pombali* Alves et al., 2006. Finally, the *didactylus* group (Izecksohn, 1971) (Pie et al., 2013—clade 3 *sensu* Clemente-Carvalho et al., 2011) essentially corresponds to the previously recognized genus *Psyllophryne* Izecksohn, 1971 known as “flea toads” and includes species with distinctly leptodactyliform body shapes when compared to the rest of the genus (i.e., *B. hermogenesi* ((Giaretta & Sawaya, 1998), and *B. didactylus* (Izecksohn, 1971)). The phylogenetic study by Clemente-Carvalho et al. (2011) based on two nuclear and three mitochondrial genes indicated considerable uncertainty in the relationships among *Brachycephalus* species, but most analyses were largely consistent with the monophyly of the three above-mentioned groups. (Another recent study reanalyzed essentially the same dataset as Clemente-Carvalho et al. (2011) as part of a large-scale phylogeny for Brachycephaloidea (Padial, Grant & Frost, 2014) and suggested that the *didactylus* and *ephippium* groups were not monophyletic, but that study only involved the analyses of the concatenated dataset and therefore did not account for the interlocus conflicts identified by Clemente-Carvalho et al. (2011).) Moreover, geometric morphometrics of cranial morphology showed that the first relative warps (which explained 59.7% of the variation in the dataset) clearly separated species from the *ephippium* group from the other two species groups, which were in turn discriminated in the second relative warps (which explained 18.8% of the variation in the dataset) (Clemente-Carvalho et al., 2011). Finally, each of the three species groups has been shown to display consistent differences in their climatic niches and, therefore, they seem to have evolved into distinct regions in climatic space (Pie et al., 2013). Other species that had not been included in the phylogeny of Clemente-Carvalho et al. (2011) can be tentatively assigned to each species group as follows: *B. bufonoides* Miranda-Ribeiro, 1920, *B. margaritatus* Pombal & Izecksohn, 2011, *B. guarani* Clemente-Carvalho et al., 2012, and *B. crispus* (Condez et al., 2014) could be assigned to the *ephippium* group based on the presence of dorsal dermal ossification (Pombal, 2010; Pombal & Izecksohn, 2011; Clemente-Carvalho et al., 2012; Condez et al., 2014; as opposed to the lack of dermal ossification in the *pernix* group—Pombal, Wistuba & Bornschein, 1998; Ribeiro et al., 2005; Alves et al., 2006) and/or by their bufoniform body shape (Pombal, 2010; Pombal & Izecksohn, 2011; Clemente-Carvalho et al., 2012; Condez et al., 2014; as opposed to the leptodactyliform body shape of the species in the *didactylus* group—Izecksohn, 1971; Giaretta & Sawaya, 1998; Napoli et al., 2011); *B. tridactylus* Garey et al., 2012 could be assigned to the *pernix* group based on the absence of dorsal dermal ossification and its bufoniform body shape (Garey et al., 2012, as opposed to the leptodactyliform body shape of the species in the *didactylus* group); finally, *B. pulex* (Napoli et al., 2011) could be assigned to the *didactylus* group because it shares several characteristics with *B. hermogenesi* and *B. didactylus*, such as a leptodactyliform body shape (as opposed to the bufoniform body shape of the species in the *pernix* and *ephippium* groups), a dark brown x-shaped mark on dorsum, and short, pointed snout with a rounded tip in dorsal view Napoli et al., 2011. There is one additional species whose validity still needs to be confirmed (i.e., *B. atelopoide* Miranda-Ribeiro, 1920). Moreover, given that the states of the criteria indicated above are still unknown for *B. atelopoide*, we chose not to assign it to any of the three species groups at the present time. Nevertheless, it is important to note that these species groups cannot be recognized as clades at the present time and should be considered as tentative, pending explicit and comprehensive phylogenetic work. Rather, they should be considered as groups correspond to ‘aggregates of species,’ as defined in Article 6.2 of the International Code of Zoological Nomenclature.

Although the first *Brachycephalus* species was described in 1824, more than half of the currently recognized species have been described during the past 15 years, suggesting the possibility that the actual diversity in the genus is considerably underestimated. In particular, there are a number of mountain ranges in the Southern Atlantic Forest that are sufficiently high to support the cloud forest typical of the habitats of many *Brachycephalus* species and that could potentially harbor new ones. Based on this expectation, our team carried out a series of field expeditions to montane areas of the Atlantic Forest from the states of Paraná and Santa Catarina, southern Brazil, to look for potentially new species. As a result, we uncovered several new species, of which seven are described herein. None of these species had been discovered prior to our expeditions, although some of the obtained records were briefly presented in a previous study on the climatic niches of *Brachycephalus* (Pie et al., 2013). In addition to clear morphological diagnostic traits, these species tend to be isolated from one another by valleys of unsuitable habitats, essentially forming “sky islands” (Howell, 1947; see McCormack, Huang & Knowles, 2009 for a recent review). These new species increase considerably the number of described species of *Brachycephalus*, including the first ones described from the state of Santa Catarina.

## Materials and Methods

Adult specimens were anaesthetized and euthanized using 30% ethanol, fixed in 10% formalin, and preserved in 70% ethanol. Measurements were made with a micrometric eyepiece attached to a stereomicroscope. The used abbreviations for measurements were as follows: snout-vent length (SVL); head length, from tip of snout to angle of jaw (HL); head width—greatest width of head located between angles of jaw (HW); eye diameter (ED); nostril diameter (ND); interorbital distance between anterior corners of the eyes (IOD); internostril distance between inner margins of nostrils (IND); eye-nostril distance from anterior corner of the eye to posterior margin of nostril (END); thigh length (THL); and tibia length (TBL). Only adult specimens were measured.

All specimens collected for this study were deposited in the Coleção Herpetológica do Departamento de Zoologia, Universidade Federal do Paraná, Curitiba, Paraná, Brazil (DZUP) and the Museu de História Natural Capão da Imbuia, Curitiba, Paraná, Brazil (MHNCI). Additionally, we examined specimens from the following collections: Célio F.B. Haddad collection (CFBH), deposited in the Departamento de Zoologia, Universidade Estadual Paulista, Campus de Rio Claro, São Paulo, Brazil, DZUP, MHNCI, Museu Nacional (MNRJ), Rio de Janeiro, Rio de Janeiro, Brazil, Museu de Zoologia da Universidade de São Paulo (MZUSP), São Paulo, São Paulo, Brazil, and Museu de História Natural (ZUEC), Universidade Estadual de Campinas, Campinas, São Paulo, Brazil. A list of the examined specimens is provided in Appendix. Throughout this study we adopted the evolutionary species concept, which equates species with “separately evolving (segments of) metapopulation lineages” (de Queiroz, 2005), which is particularly suitable for the evolutionary scenario of population isolation in sky islands followed by divergent evolution.

Collection permits for this study were issued by the Fundação Municipal do Meio Ambiente (FUNDEMA)/Municipality of Joinville (001/11-GEMAP), Instituto Ambiental do Paraná (permit 355/11), and ICMBIO (SISBIO 10.500, 22470-2). We classified vegetation in collection sites according to the classification of Veloso, Rangel-Filho & Lima (1991). All coordinates in this study were based on datum WGS84.

## Results

**Table:** 

**Figure 1:**
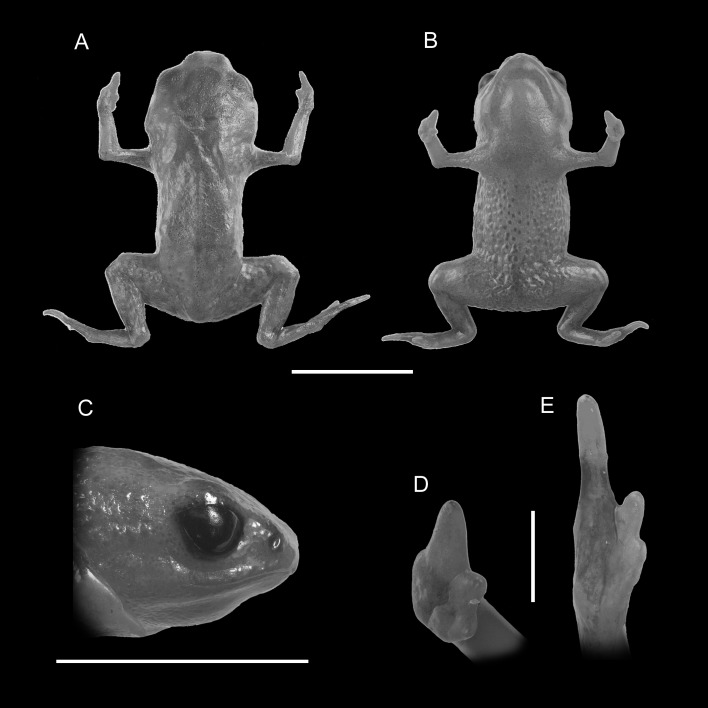
Holotype of *Brachycephalus mariaeterezae* (MHNCI 9811). (A) dorsal view of the body, (B) ventral view of the body, (C) lateral view of the head, (D) ventral view of right hand, (E) ventral view of right foot. Horizontal scale bar = 5 mm; vertical scale bars = 2 mm.

**Figure 2:**
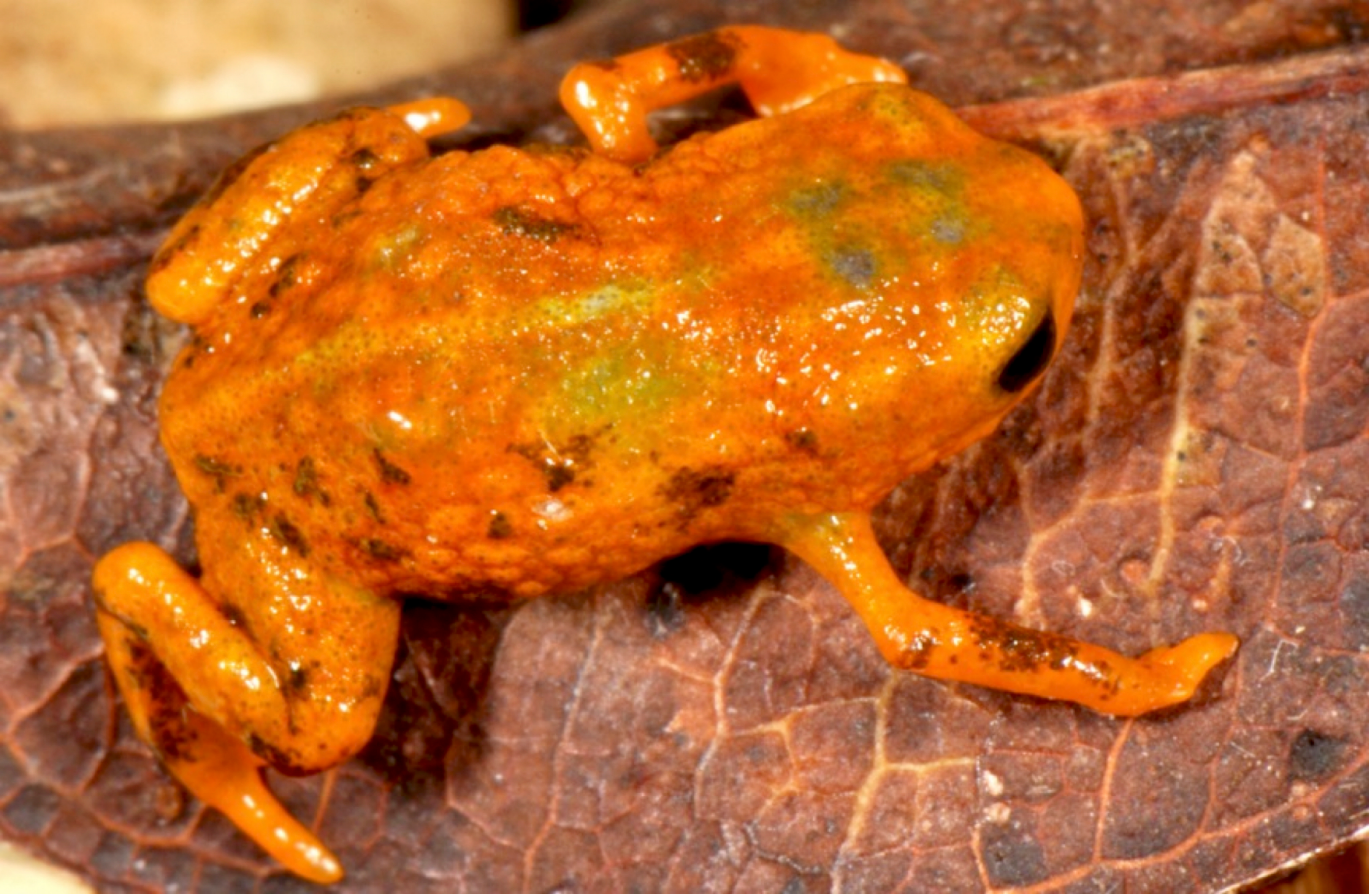
*Brachycephalus mariaeterezae* in life. The intensity of the light of the flash during photography led the light-blue coloration along their vertebral column to become less apparent.

*Brachycephalus mariaeterezae* sp. nov.
Bornschein, Morato, Firkowski, Ribeiro & Pie
(Figs. 1 and 2)
*Brachycephalus* sp. nov. 6 (Pie et al., 2013)
Urn:lsid:zoobank.org:act: 74D8D85E-BE68-4A51-AF85-DC97C564D458

### Holotype

MHNCI 9811 (ex DZUP 168— Fig. 1), adult male, collected at Reserva Particular do Patrimônio Natural Caetezal, top of the Serra Queimada (26°06′51″S, 49°03′45″W; 1,270 m a.s.l.), municipality of Joinville, state of Santa Catarina, southern Brazil, on 27 January 2011 by MRB and SAAM.

### Paratopotypes

MHNCI 9812 (ex DZUP 169, diaphanized), collected by 27 January 2011 by MRB and SAAM, DZUP 372, 393-9, nine adults collected with the holotype on 26 January 2012 by MRP, LFR and Felipe Cini, between 1,265–1,270 m a.s.l.

### Diagnosis

*Brachycephalus mariaeterezae* is distinguished from all of the species in the genus by the following combination of characters: Body robust, bufoniform, adult size SVL 10.4–13.4 mm, rough dorsum (Fig. 1); general background color yellow, with small brown spots concentrated on the sides of the body and belly and an irregular light-blue stripe on dorsum of the head and central body dorsum; small dark spots distributed irregularly throughout arms and legs; skin on dorsum of head and central body dorsum with no dermal co-ossification (Fig. 2). A representative of the *pernix* group similar to *B. ferruginus*, *B. izecksohni*, *B. pombali*, and *B. tridactylus* by its general yellow color, but all of these species lack the bluish color of the dorsum and brown spots dispersal on the body present in *B. mariaeterezae*. The new species lacks the brownish line or spots on the center of the dorsum present on some *B. ferruginus*. Additionally, *B. pombali* and *B. tridactylus* have orange body coloration. The new species is also distinguished from *B. ferruginus*, *B. izecksohni*, *B. pombali*, and *B. tridactylus* by having rough dorsum, with granular aspect, instead of a smooth dorsum. With respect to the remaining species of *pernix* group, *B. mariaeterezae* is easily distinguished from *B. brunneus* and *B. pernix* by its coloration and rough dorsum, given that *B. brunneus* are dark brown with only small yellow spots scattered on venter and *B. pernix* are yellow with brown on sides of body, and both species have smooth dorsum. The new species lacks the dermal co-ossification characteristic of species of the *ephippium* group (Clemente-Carvalho et al., 2011; Pie et al., 2013), and the bufoniform shape and larger body size of the new species distinguish it from those in the *didactylus* group (Clemente-Carvalho et al., 2011; Pie et al., 2013), which are smaller (SVL = 8–10 mm) and have a leptodactyliform body shape.

### Description of holotype

Body robust, bufoniform; head slightly wider than long; head length 33% of snout-vent length; snout short, with length almost equal to eye diameter, rounded in dorsal and lateral views; nostrils protuberant, directed anterolaterally; canthus rostralis indistinct; lips nearly sigmoid; loreal region slightly concave; eye slightly protuberant in dorsal and lateral views; ED 39% of head length; tympanum indistinct; vocal sac not expanded externally; vocal slits present; tongue longer than wide, with posterior half not adherent to floor of mouth; choanae relatively small and round; vomerine odonthophores absent; arm and forearm relatively slender; arm approximately as long as forearm; tip of finger I and II slightly rounded, tip of finger III pointed; finger I and IV very small, vestigial; relative lengths of fingers IV < I < II < II; subarticular tubercles absent; inner and outer metacarpal tubercles absent; legs short, moderately robust; thigh length 37% of snout-vent length, tibia length 86% of thigh length; toes II–III short, relatively distinct; toes I and V externally absent; relative length of toes II < III < IV; subarticular tubercles and inner metatarsal tubercles absent; outer metatarsal tubercle distinct, large, ovoid; rough dorsum, without co-ossifications; glandular warts large, circular to oval, present widely spaced in dorsum and legs; head and arms smooth; sides of the body granular; large glandular warts circular and juxtaposed on the sides of the body; belly granular; circular glandular warts juxtaposed, distributed from belly to legs and arms, chin smooth.

### Coloration of Holotype

In life, dorsum, head, sides of the body, dorsal region of arms, thighs, and legs yellow; brown spots, of irregular shape and varied sizes, distributed across the dorsum, arms, thighs, and legs; light-blue spots, irregular in size and shape, on the dorsal surface of the head; belly, chin, ventral portion of arms, thighs, legs, and ventral regions of hand and feet yellow; small brown spots, nearly circular, on the belly; irregular brown spots around nostrils; iris black (see Fig. 2). In preservative, general color pale cream; sides of the body pale cream with dark brown spots; pale grayish coalescent spots and small dots on belly.

### Measurements of holotype

SVL = 11.2 mm, HL = 3.6 mm, HW = 4.1 mm, ED = 1.4 mm, ND = 0.2 mm, IOD = 2.4 mm, IND = 1.3 mm, END = 0.7 mm, THL = 4.1 mm, TBL = 3.6 mm.

### Variation in type series

Measurements and proportions of 10 adults are given in Tables 1 and 2. The intensity and the width of the light-blue stripe along the dorsal region may vary among individuals, as well as the number and size of dark spots on their dorsum (Figure S1).

**Table 1:** Measurements (mm) of the type series of *Brachycephalus mariaeterezae*.

Trait	$\bar {X}$	SD	Range
SVL	11.27	0.97	10.4–13.4
HL	3.72	0.13	3.6–3.9
HW	4.36	0.35	3.9–5.0
ED	1.44	0.12	1.3–1.6
ND	0.17	0.02	0.2–0.2
IOD	2.41	0.19	2.2–2.9
IND	1.35	0.08	1.3–1.6
END	0.68	0.07	0.6–0.8
THL	4.08	0.44	3.3–5.0
TBL	3.65	0.19	3.4–4.1

**Notes.**

$\bar {X}$ mean SD standard deviation

*N* = 10 individuals.

Character abbreviations listed in the Material and Methods.

**Table 2:** Proportions of the type series of *Brachycephalus mariaeterezae*.

	$\bar {X}$	SD	Range
THL/SVL	36%	3%	31–39%
TBL/THL	90%	7%	83–104%
HL/SVL	33%	2%	29–36%
ED/HL	39%	2%	36–42%
HW/HL	117%	7%	104–130%

**Notes.**

$\bar {X}$ mean SD standard deviation

Character abbreviations listed in the Material and Methods.

### Etymology

The specific epithet honors Maria Tereza Jorge Pádua, tireless environmentalist responsible for the implementation of many conservation initiatives in Brazil.

### Distribution

*Brachycephalus mariaeterezae* is known only from the type locality.

### Remarks

Individuals of *B. mariaeterezae* were found on leaf litter of a cloud forest (“Floresta Ombrófila Densa Altomontana”) between 1,265–1,270 m a.s.l. During two visits to the type locality, we found individuals of *B. mariaeterezae* hidden in the leaf litter, some of which vocalizing. The species is known from a restricted area that is under a variety of sources of perturbation, including deforestation, fire, and cattle farming—even though it theoretically should have been protected given its current status as a private reserve (“Reserva Particular do Patrimônio Natural”). Therefore, the conservation of this locality is necessary for the survival of this species.

**Table:** 

**Figure 3:**
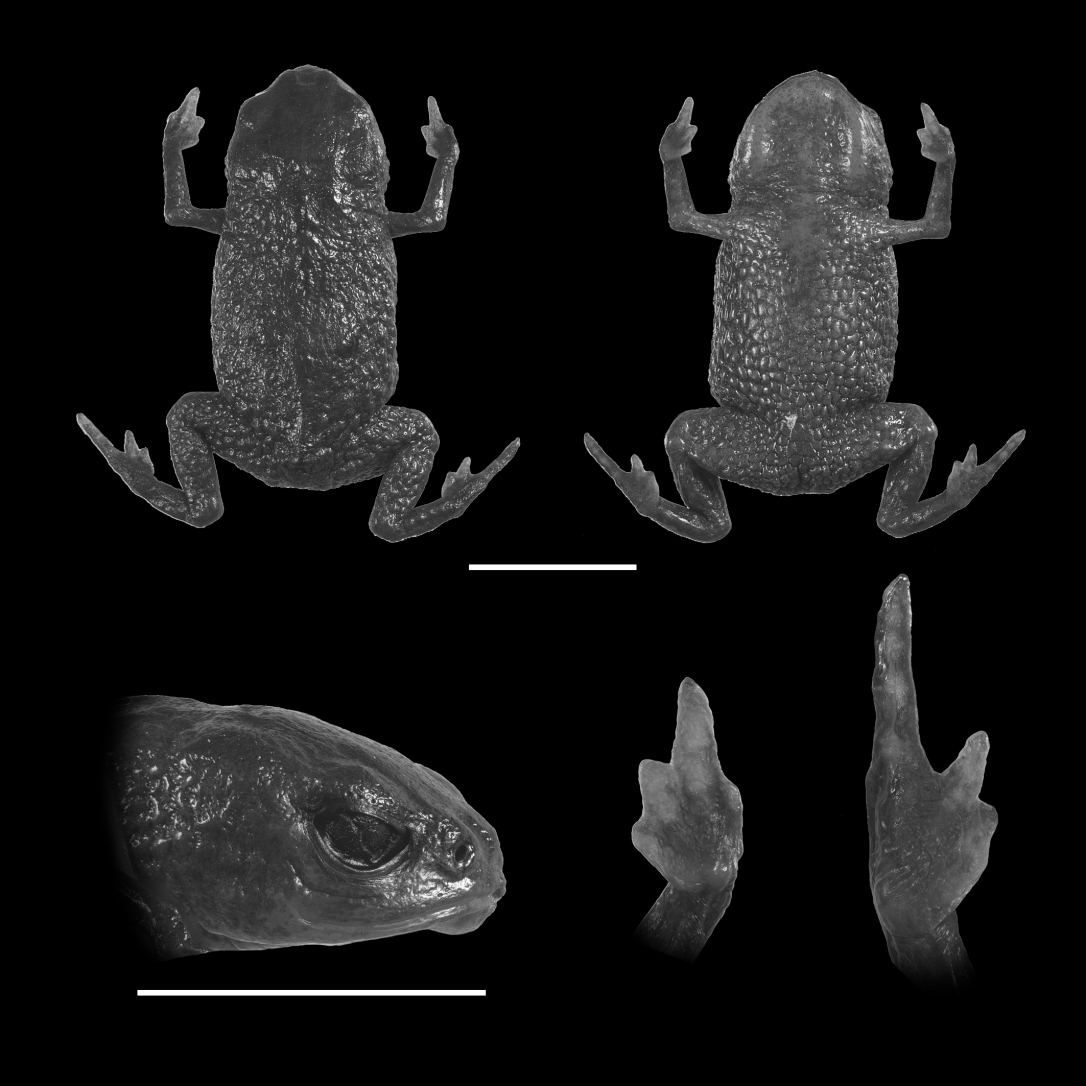
Holotype of *Brachycephalus olivaceus* (MHNCI 9813). (A) dorsal view of the body, (B) ventral view of the body, (C) lateral view of the head, (D) ventral view of left hand, (E) ventral view of right foot. Horizontal scale bar = 5 mm; vertical scale bars = 2 mm.

**Figure 4:**
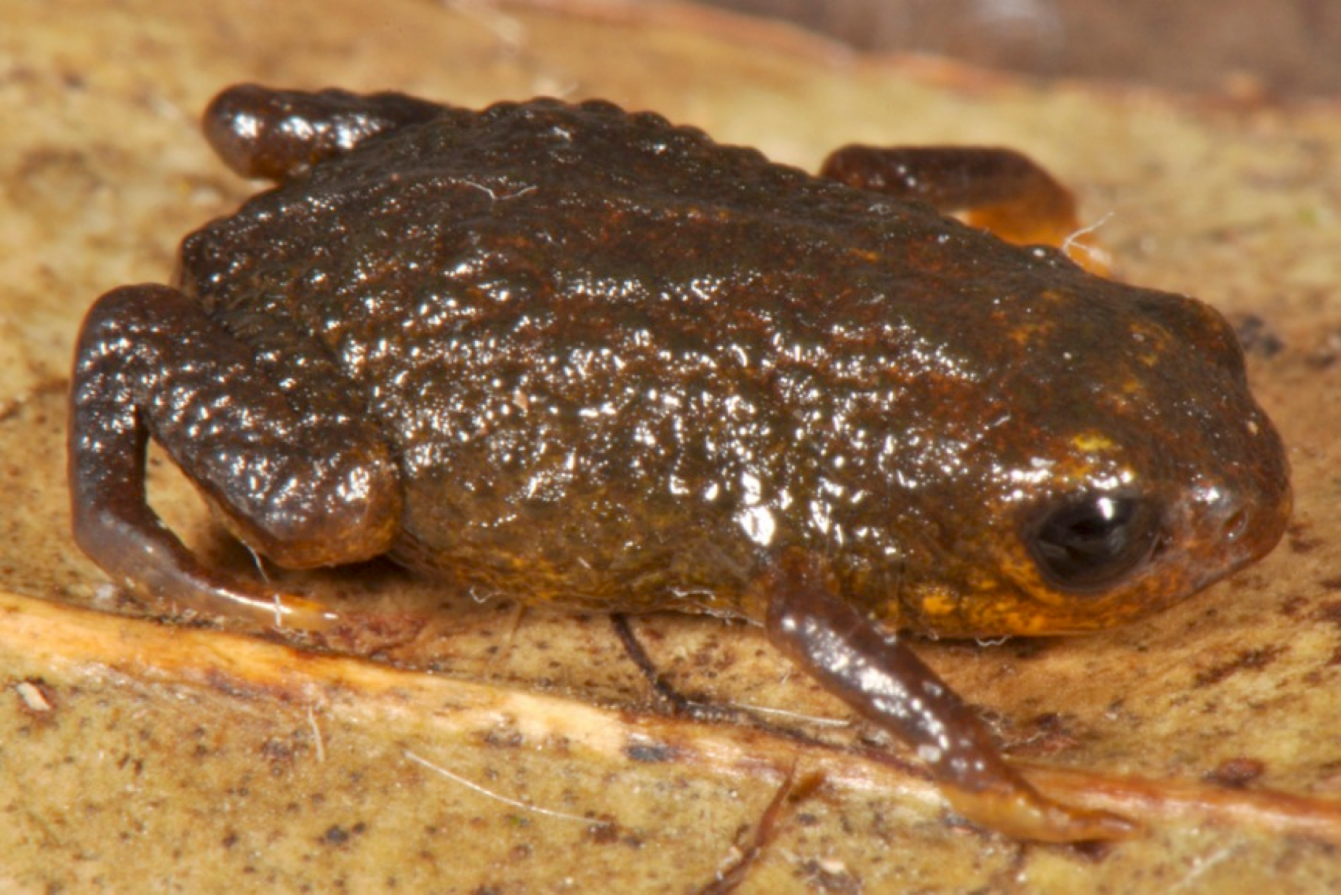
*Brachycephalus olivaceus* in life.

*Brachycephalus olivaceus* sp. nov.
Bornschein, Morato, Firkowski, Ribeiro & Pie
(Figs. 3 and 4)
*Brachycephalus* sp. nov. 7 (Pie et al., 2013)
Urn:lsid:zoobank.org:act: 6BBEE98B-0296-415D-BEB2-F23031FE8BF6

### Holotype

MHNCI 9813 (ex DZUP 170— Fig. 3), adult female collected at the base of the Serra Queimada (26°04′57″S, 49°03′59″W; 985 m a.s.l.), municipality of Joinville, state of Santa Catarina, southern Brazil, on 23 January 2011 by MRB and SAAM.

### Paratopotype

DZUP 371, collected by MRP, LFR and Felipe Cini on 26 January 2012.

### Paratypes

MHNCI 9814–5 (ex DZUP 163-4), adults collected at Castelo dos Bugres (26°13′59″S, 49°03′13″W; 800–835 m a.s.l.), municipality of Joinville, state of Santa Catarina, southern Brazil, on 19 January 2011 by MRB and SAAM; MHNCI 9816-8 (ex DZUP 165-7), adults collected in the same locality by the same collectors on 22 January 2011.

### Diagnosis

*Brachycephalus olivaceus* is distinguished from all of the species in the genus by the following combination of characters: Body robust, bufoniform, adult SVL 9.4–12.9 mm, rough dorsum (Fig. 3); general coloration predominantly dark-green to brown; skin on dorsum of head and central body dorsum with no dermal co-ossification (Fig. 4). A representative of the *pernix* group, its rugose body dorsum is similar to that of *B. mariaeterezae* (as opposed to the smooth dorsum of *B. izecksohni*, *B. brunneus*, *B. pernix*, *B. ferruginus*, *B. pombali*, and *B. tridactylus*). It also differs from *B. mariaeterezae* due to its rugose head dorsum (which is smooth in *B. izecksohni*, *B. brunneus*, *B. pernix*, *B. ferruginus*, and *B. mariaeterezae*). The predominantly dark-green to brown dorsum of *B. olivaceus* is distinct from all *Brachycephalus* species. The new species lacks the dermal co-ossification characteristic of species of the *ephippium* group, and the bufoniform shape and larger body size of the new species distinguish it from those in the *didactylus* group, which are smaller (SVL = 8–10 mm) and have a leptodactyliform body shape.

### Description of Holotype

Body robust, bufoniform; head slightly wider than long; head length 36% of snout-vent length; snout short, with length almost equal to eye diameter, rounded in dorsal and lateral views; nostrils protuberant, directed anterolaterally; canthus rostralis indistinct; lips nearly sigmoid; loreal region slightly concave; eye slightly protuberant in dorsal and lateral views; ED 26% of head length; tympanum indistinct; vocal sac not expanded externally; vocal slits present; tongue longer than wide, with posterior half not adherent to floor of mouth; choanae relatively small and round; vomerine odonthophores absent; arm and forearm relatively slender; arm approximately as long as forearm; tip of finger I and II slightly rounded, tip of finger III pointed; finger I and IV very small, vestigial; relative lengths of fingers IV < I < II < II; subarticular tubercles absent; inner and outer metacarpal tubercles absent; legs short, moderately robust; thigh length 32% of snout-vent length, tibia length 93% of thigh length; toes II–III short, relatively distinct; toes I and V externally absent; relative length of toes II <III < IV; subarticular tubercles and inner metatarsal tubercles absent; outer metatarsal tubercle distinct, large, ovoid; rugose dorsum without co-ossifications; glandular warts large, circular or oval, widely spaced on dorsum and legs; numerous small glandular warts on head and arms; sides of the body granular; circular glandular warts not so juxtaposed on the sides of the body; belly granular; circular glandular warts juxtaposed, distributed from belly to legs; chin and arms with small glandular warts.

### Coloration of holotype

In life, dorsum, head, sides of the body, arms, legs, and thighs dark green; throat, belly, ventral region of arms, legs, thighs, fingers, and toes with small irregular orange spots, not juxtaposed, at irregular positions; iris black (Fig. 4). In preservative, general color dark brown; tips of fingers and toes slightly become less pigmented.

### Measurements of holotype

SVL = 12.9 mm, HL = 4.6 mm, HW = 4.9 mm, ED = 1.2 mm, ND = 0.2 mm, IOD = 2.6 mm, IND = 1.4 mm, END = 0.8 mm, THL = 4.1 mm, TBL = 3.9 mm.

### Variation in type series

Measurements and proportions of seven adults are given in Tables 3 and 4. The dark-green coloration might extend into the fingers and toes in some individuals. The belly coloration might vary in the extent of the orange coloration, being most concentrated around the ventral region of the chin and center of the belly.

**Table 3:** Measurements (mm) of the type series of *Brachycephalus olivaceus*.

Trait	$\bar {X}$	SD	Range
SVL	10.50	1.32	9.4–12.9
HL	3.62	0.52	3.0–4.6
HW	4.02	0.49	3.6–4.9
ED	1.14	0.15	1.0–1.5
ND	0.17	0.03	0.2–0.2
IOD	2.07	0.32	1.8–2.6
IND	1.14	0.12	1.0–1.4
END	0.61	0.08	0.6–0.8
THL	3.66	0.35	3.1–4.1
TBL	3.15	0.38	2.9–3.9

**Notes.**

$\bar {X}$ mean SD standard deviation

*N* = 17 specimens.

Character abbreviations listed in the Material and Methods.

**Table 4:** Proportions of the type series of *Brachycephalus olivaceus*.

	$\bar {X}$	SD	Range
THL/SVL	35%	2%	32–38%
TBL/THL	86%	4%	81–93%
HL/SVL	34%	1%	32–36%
ED/HL	32%	3%	26–36%
HW/HL	112%	6%	106–124%

**Notes.**

$\bar {X}$ mean SD standard deviation

Character abbreviations listed in the Material and Methods.

### Etymology

The specific epithet is from the Latin olivaceus (“olive-colored”), in reference to the general dark green coloration of the new species.

### Distribution

*Brachycephalus olivaceus* is known only from two nearby localities—the base of the Serra Queimada and Castelo dos Bugres, in the municipality of Joinville, in the northeastern state of Santa Catarina.

### Remarks

Individuals of the new species were found on leaf litter at montane forest (“Floresta Ombrófila Densa Montana”) between 800–985 m a.s.l., near the location where *B. mariaeterezae* was uncovered. Individuals were active by day, and calling males were found in the leaf litter. The species is known from a restricted area that is under a variety of sources of perturbation, including deforestation and cattle farming. Therefore, the conservation of these localities is necessary for the survival of this species.

**Table:** 

**Figure 5:**
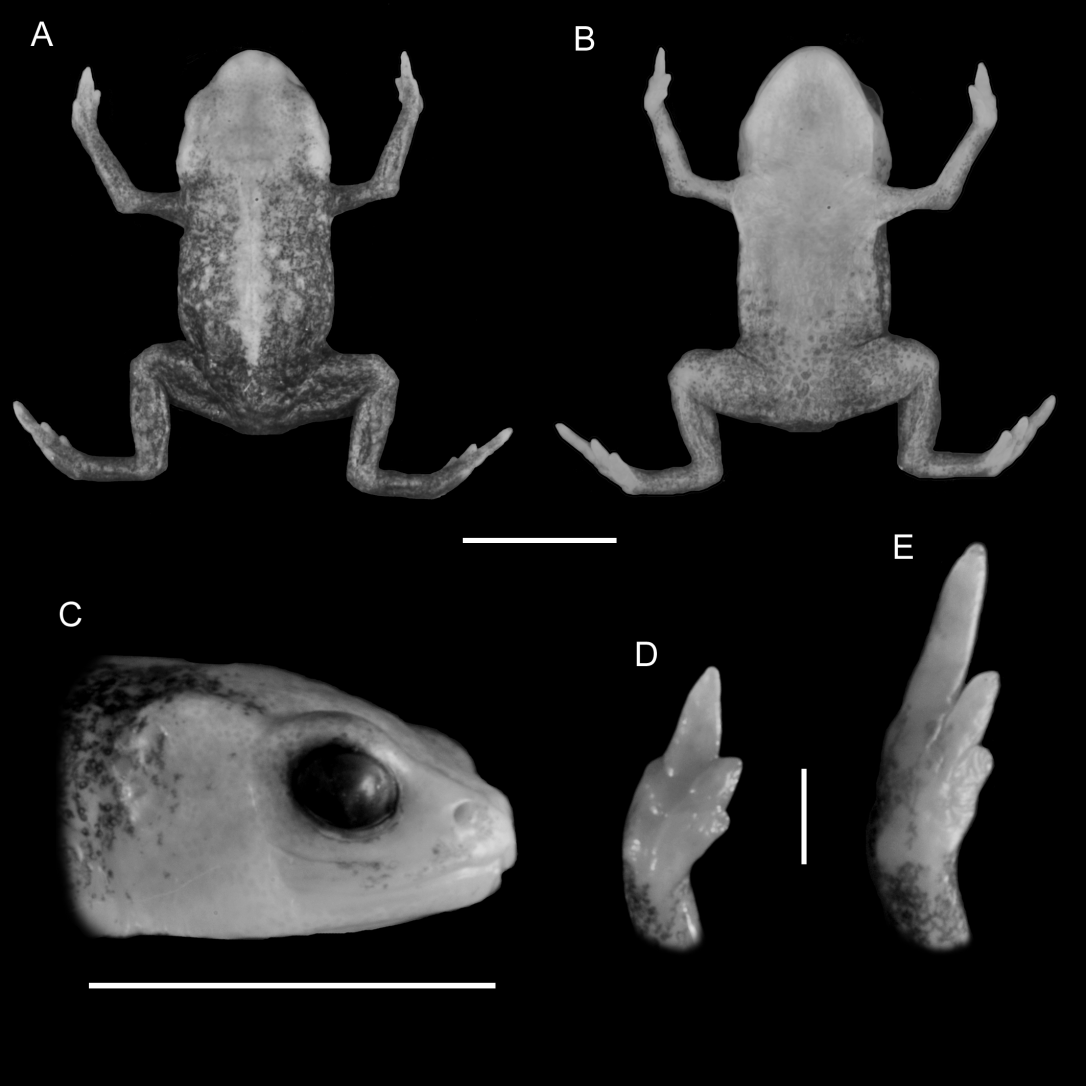
Holotype of *Brachycephalus auroguttatus* (DZUP 375). (A) dorsal view of the body, (B) ventral view of the body, (C) lateral view of the head, (D) ventral view of right hand, (E) ventral view of right foot. Horizontal scale bar = 5 mm; vertical scale bars = 2 mm.

**Figure 6:**
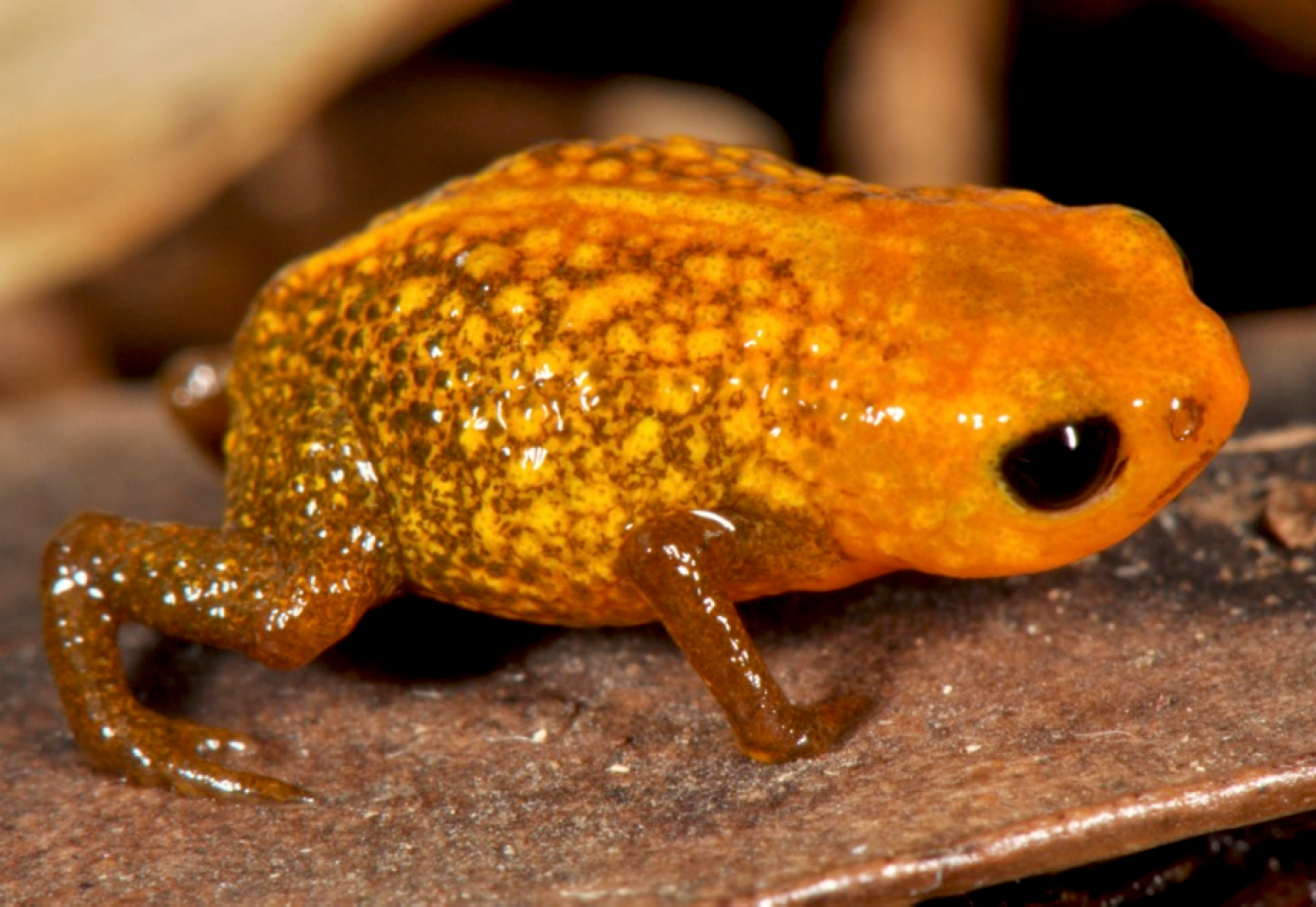
*Brachycephalus auroguttatus* in life.

*Brachycephalus auroguttatus* sp. nov.
Ribeiro, Firkowski, Bornschein & Pie
(Figs. 5 and 6)
Urn:lsid:zoobank.org:act: DB8A686E-4C9E-467D-8BF1-B841B21133AB

### Holotype

DZUP 375 (Fig. 5), adult male collected at Pedra da Tartaruga (26°00′21″S, 48°55′25″W; 1,070 m a.s.l.), municipality of Garuva, state of Santa Catarina, southern Brazil, on 4 February 2012 by LFR, Marcelo Leandro Brotto and Lucas Mendes.

### Paratopotypes

DZUP 373-4, 376-85, 387-89, 15 adults collected with the holotype by LFR, Marcelo Leandro Brotto and Lucas Mendes, between 1,070–1,100 m a.s.l.

### Diagnosis

*Brachycephalus auroguttatus* is distinguished from all of the species in the genus by the following combination of characters: Body robust, bufoniform, adult SVL 9.0–13.6 mm, rough dorsum (Figs. 5 and 6); dorsal coloration shifting from bright yellow on the head with increasingly more brown toward the posterior region of the body, legs and arms, with yellow spots along the back; skin on dorsum of head and central body dorsum with no dermal co-ossification (Fig. 6). A representative of the *pernix* group, its rugose body dorsum is similar to that of *B. olivaceus* and *B. mariaeterezae*—as opposed to the smooth dorsum of *B. izecksohni*, *B. brunneus*, *B. pernix*, *B. ferruginus*, *B. pombali*, and *B. tridactylus*. However, its dorsal coloration shifting from bright yellow on the head with increasingly more brown toward the posterior region of the body is distinct from the dark green general coloration of *B. olivaceus* and the blue stripe and overall yellow dorsal coloration of *B. mariaeterezae*. The new species has a yellow stripe on its back, similar to *B. pombali*, but differs from that species in its general coloration, given that *B. pombali* is mostly orange throughout its dorsum. On the other hand, the new species is similar to *B. pombali* in its overall orange coloration, but *B. pombali* lacks the yellow stripe along the back of the new species. The new species shares with *B. pernix* and *B. tridactylus* the dark spots along the sides of the body, but differs from those species by its overall coloration, which is yellow without a shift to brown towards the posterior region of the body in both *B. pernix* and *B. tridactylus*. The new species lacks the dermal co-ossification characteristic of species of the *ephippium* group, and the bufoniform shape and larger body size of the new species distinguish it from those in the *didactylus* group, which are smaller (SVL = 8–10 mm) and have a leptodactyliform body shape.

### Description of holotype

Body robust, bufoniform; head slightly wider than long; head length 33% of snout-vent length; snout short, with length almost equal to eye diameter, rounded in dorsal and lateral views; nostrils not protuberant, directed anterolaterally; canthus rostralis indistinct; lips nearly sigmoid; loreal region slightly concave; eye slightly protuberant in dorsal and lateral views; ED 33% of head length; tympanum indistinct; vocal sac not expanded externally; vocal slits present; tongue longer than wide, with posterior half not adherent to floor of mouth; choanae relatively small and round; vomerine odonthophores absent; arm and forearm relatively slender; arm approximately as long as forearm; tip of finger I and II slightly rounded, tip of finger III pointed; finger I and IV very small, vestigial; relative lengths of fingers IV < I <II < III; subarticular tubercles absent; inner and outer metacarpal tubercles absent; legs short, moderately robust; thigh length 35% of snout-vent length, tibia length 87% of thigh length; toes II–III short, relatively distinct; toes I and V externally absent; relative length of toes II < III < IV; subarticular tubercles and inner metatarsal tubercles absent; outer metatarsal tubercle distinct, large, ovoid; dorsum granular, without co-ossifications; glandular warts circular or oval, juxtaposed on dorsum and legs; small glandular warts on arms; head smooth; sides of the body granular; large glandular warts circular and not so juxtaposed on the sides of the body; belly granular; circular glandular warts juxtaposed, distributed from belly to legs; chin and arms smooth.

### Coloration of holotype

In life, dorsal region of head and along the vertebral column forming a yellow stripe; remaining regions of body with a mixture of brown and yellow, with increasingly more brown towards the posterior region of the body, including sides of the body; arms, legs, thighs, hands, and feet, in dorsal view, predominantly brown; belly, arms, thighs and legs, in ventral view, with a mixture of orange and brown; outline of the nostrils and upper region of mouth with fine lines of brown; iris black (Fig. 6). In preservative, originally orange and yellow regions become pale cream, whereas brown regions become slightly faded.

### Measurements of holotype

SVL = 12.6 mm, HL = 4.1 mm, HW = 4.7 mm, ED = 1.4 mm, ND = 0.2 mm, IOD = 2.4 mm, IND = 1.4 mm, END = 0.7 mm, THL = 4.4 mm, TBL = 3.9 mm.

### Variation in type series

Measurements and proportions are given in Tables 5 and 6. The brown coloration found in legs and arms might extend slightly into nearby regions of the body, particularly in the case of the legs.

**Table 5:** Measurements (mm) of the type series of *Brachycephalus auroguttatus*.

Trait	$\bar {X}$	SD	Range
SVL	11.49	1.27	9.0–13.6
HL	3.84	0.4	3.1–4.4
HW	4.51	0.42	3.9–5.1
ED	1.26	0.11	1.1–1.5
ND	0.18	0.02	0.2–0.2
IOD	2.32	0.23	2.0–2.8
IND	1.31	0.12	1.2–1.6
END	0.66	0.08	0.5–0.8
THL	4.16	0.4	3.4–4.9
TBL	3.61	0.41	2.7–4.1

**Notes.**

$\bar {X}$ mean SD standard deviation

*N* = 17 specimens.

Character abbreviations listed in the Material and Methods.

**Table 6:** Proportions of the type series of *Brachycephalus auroguttatus*.

	$\bar {X}$	SD	Range
THL/SVL	36%	2%	33–40%
TBL/THL	87%	5%	79–100%
HL/SVL	33%	2%	29–38%
ED/HL	33%	3%	30–44%
HW/HL	118%	7%	107–130%

**Notes.**

$\bar {X}$ mean SD standard deviation

Character abbreviations listed in the Material and Methods.

### Etymology

The specific epithet is from the Latin aurum (“gold”) and gutta (“drop,” “spot,” “speck”), in reference to the golden spots found throughout the dorsum and sides of the body of the new species.

### Distribution

*Brachycephalus auroguttatus* is known only from the type locality.

### Remarks

Individuals of the new species were found on leaf litter of a cloud forest (“Floresta Ombrófila Densa Altomontana”) between 1,070–1,100 m a.s.l. During a visit to the type locality, we found individuals of *B. auroguttatus* hidden in the leaf litter, some of which vocalizing.

**Table:** 

**Figure 7:**
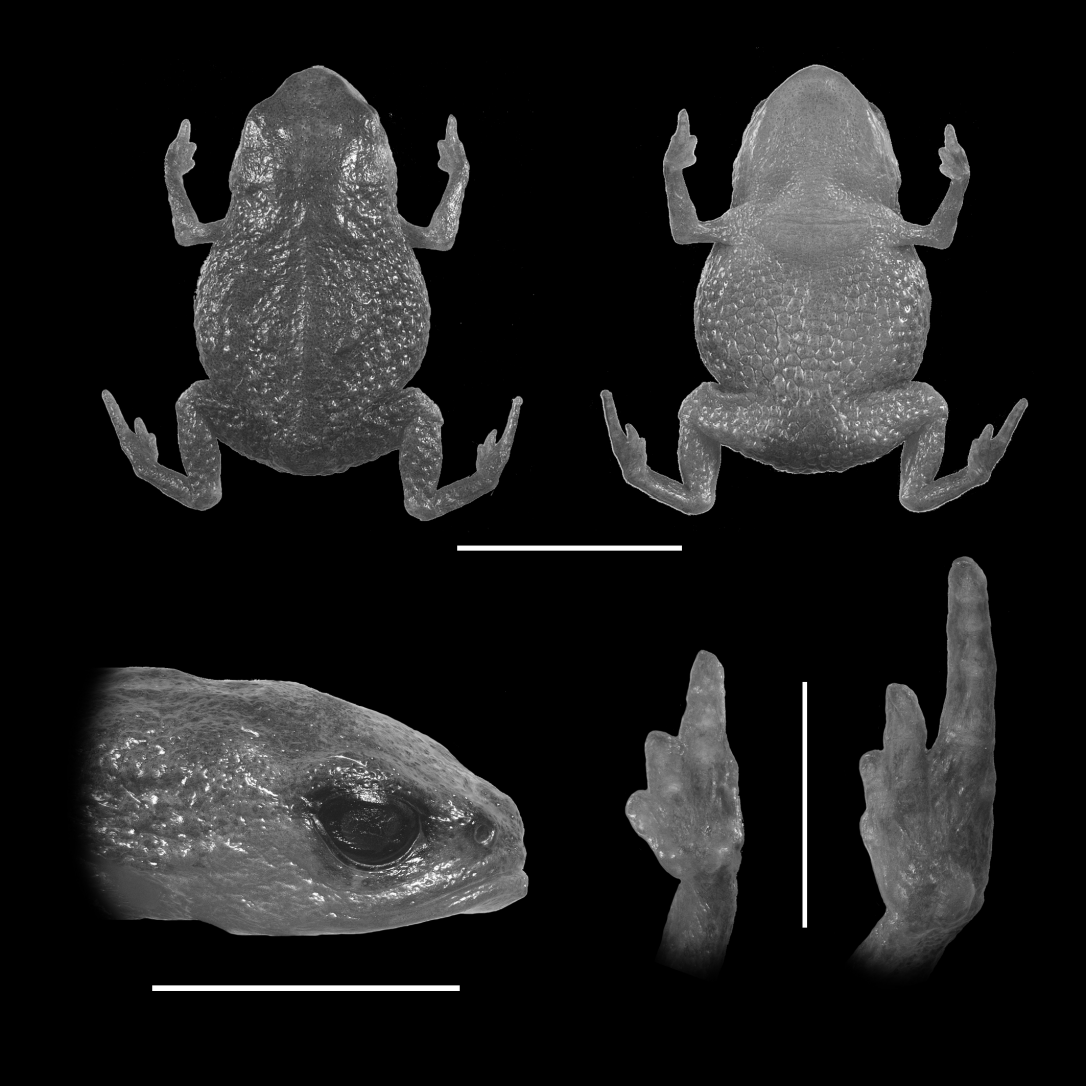
Holotype of *Brachycephalus verrucosus* (MHNCI 9819). (A) dorsal view of the body, (B) ventral view of the body, (C) lateral view of the head, (D) ventral view of left hand, (E) ventral view of left foot. Horizontal scale bar = 5 mm; vertical scale bars = 2 mm.

**Figure 8:**
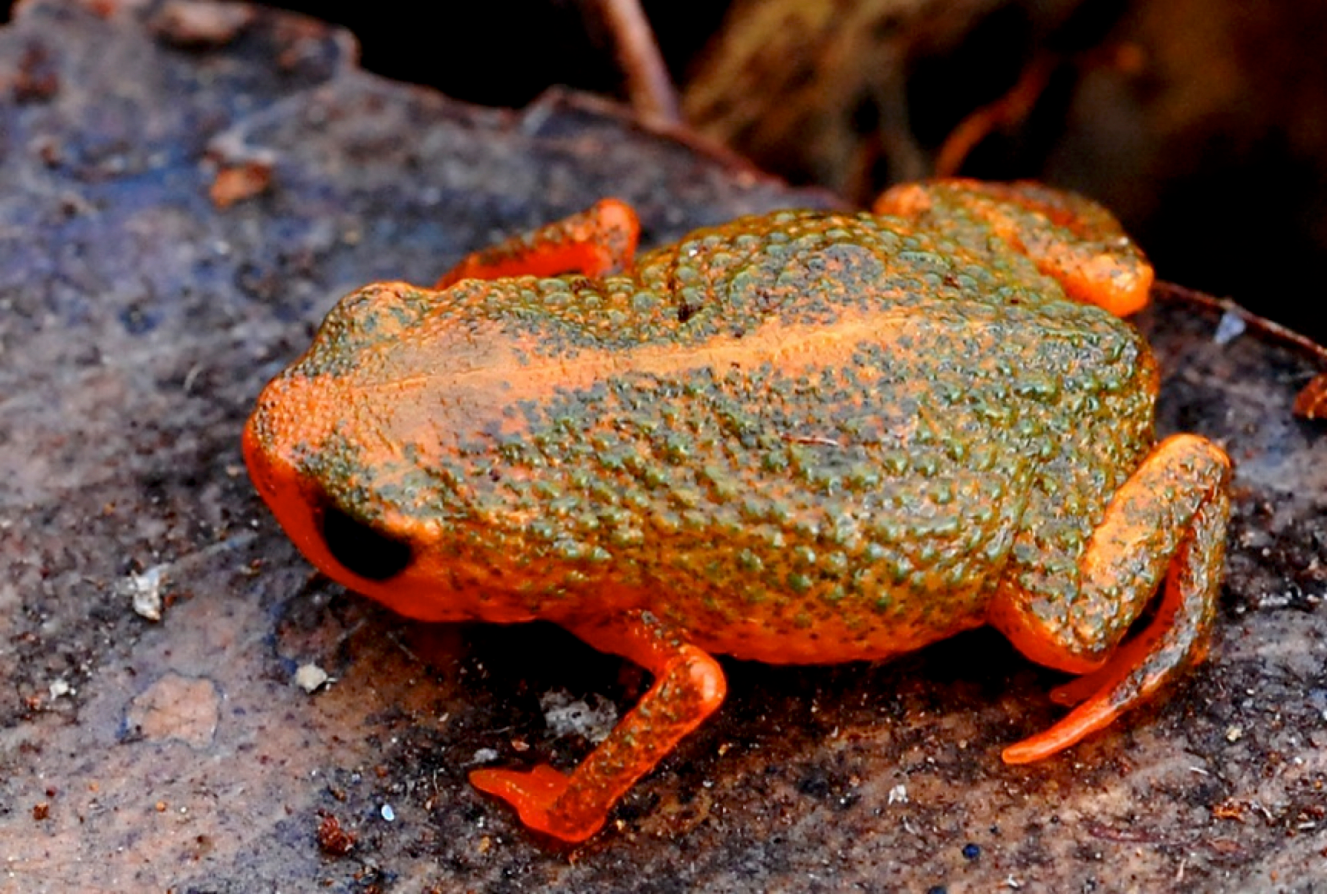
*Brachycephalus verrucosus* in life.

*Brachycephalus verrucosus* sp. nov.
Ribeiro, Firkowski, Bornschein & Pie
(Figs. 7 and 8)
*Brachycephalus* sp. nov. 8 (Pie et al., 2013)
Urn:lsid:zoobank.org:act: 2CCC4A91-65E8-485F-B864-25CA9589F48F

### Holotype

MHNCI 9819 (ex DZUP 161— Fig. 7), adult female collected at Morro da Tromba (26°12′44″S, 48°57′29″W; 900 m a.s.l.), municipality of Joinville, state of Santa Catarina, southern Brazil, on 25 January 2011, by MRB and SAAM.

### Paratopotypes

MHNCI 9820 (ex DZUP 162) adult collected with the holotype, by MRB and SAAM; DZUP 464–78, 14 adults collected by Marcelo Leandro Brotto and LFR, on 05 February 2010, between 455–945 m a.s.l.

### Diagnosis

*Brachycephalus verrucosus* is distinguished from all of the species in the genus by the following combination of characters: Body robust, bufoniform, adult SVL 9.6–13.2 mm, very rough dorsum (Fig. 7); general coloration of dorsum light-green with a thin orange stripe along most of the vertebral column in some individuals; skin on dorsum of head and central body dorsum with no dermal co-ossification (Fig. 8). A representative of the *pernix* group, it is most similar to *B. olivaceus* among all other congeners due to the overall green coloration in their dorsum, yet the orange coloration of its belly differs from the yellow and green coloration of the belly of *B. olivaceus*. Also, the coloration of the dorsum of *B. olivaceus* is dark green, whereas the dorsal coloration of the new species is of a lighter overall color due to the alternation of small yellow and green spots. The rugose body dorsum of the new species is similar to that of *B. mariaeterezae*, *B. olivaceus* and *B. auroguttatus*, as opposed to the smooth dorsum of *B. izecksohni*, *B. brunneus*, *B. pernix*, *B. ferruginus*, *B. pombali*, and *B. tridactylus.*The new species lacks the dermal co-ossification characteristic of species of the *ephippium* group, and the bufoniform shape and larger body size of the new species distinguish it from those in the *didactylus* group, which are smaller (SVL = 8–10 mm) and have a leptodactyliform body shape.

### Description of holotype

Body robust, bufoniform; head slightly wider than long; head length 33% of snout-vent length; snout short, with length almost equal to eye diameter, rounded in dorsal and lateral views; nostrils slightly protuberant, directed anterolaterally; canthus rostralis indistinct; lips nearly sigmoid; loreal region slightly concave; eye slightly protuberant in dorsal and lateral views; ED 32% of head length; tympanum indistinct; vocal sac not expanded externally; vocal slits present; tongue longer than wide, with posterior half not adherent to floor of mouth; choanae relatively small and round; vomerine odonthophores absent; arm and forearm relatively slender; arm approximately as long as forearm; tip of finger I and II slightly rounded, tip of finger III pointed; finger I and IV very small, vestigial; relative lengths of fingers IV < I < II < II; subarticular tubercles absent; inner and outer metacarpal tubercles absent; legs short, moderately robust; thigh length 33% of snout-vent length, tibia length 81% of thigh length; toes II–III short, relatively distinct; toes I and V externally absent; relative length of toes II < III < IV; subarticular tubercles and inner metatarsal tubercles absent; outer metatarsal tubercle distinct, large, ovoid; dorsum granular, without co-ossifications; glandular warts large, circular, oval or elongate, juxtaposed on dorsum and legs; numerous small glandular warts on head and arms; sides of the body granular; large glandular warts circular and oval, juxtaposed on the sides of the body; belly granular; circular glandular warts juxtaposed, distributed from belly to legs; chin and arms smooth.

### Coloration of holotype

In life, head, dorsum of the body, dorsal region of arms, thighs, and legs light, light green; lateral regions of the body, belly, chin, ventral portion of arms, legs, thighs, hands, and feet green near the dorsum becoming yellow towards the venter; spots around nostrils irregular, yellowish green; iris black (Fig. 8). In preservative, pale cream with brown in the center of the vertebral column.

### Measurements of holotype

SVL = 10.9 mm, HL = 3.6 mm, HW = 4.0 mm, ED = 1.2 mm, ND = 0.2 mm, IOD = 2.0 mm, IND = 1.2 mm, END = 0.6 mm, THL = 3.7 mm, TBL = 3.0 mm.

### Variation in type series

Measurements and proportions of 14 adults are given in Tables 7 and 8. There is variation among individuals in the extent of yellow surrounding the light-green regions, at times leading to a orange stripe along their vertebral column and on the dorsal region of the head.

**Table 7:** Measurements (mm) of the type series of *Brachycephalus verrucosus*.

Trait	$\bar {X}$	SD	Range
SVL	11.35	0.99	9.6–13.2
HL	3.72	0.39	3.1–4.4
HW	4.38	0.33	4.0–5.0
ED	1.26	0.09	1.1–1.5
ND	0.20	0.03	0.2–0.3
IOD	2.30	0.16	2.1–2.6
IND	1.26	0.09	1.2–1.4
END	0.66	0.07	0.6–0.8
THL	3.95	0.28	3.4–4.7
TBL	3.53	0.30	3.0–4.0

**Notes.**

$\bar {X}$ mean SD standard deviation

*N* = 14 specimens.

Character abbreviations listed in the Material and Methods.

**Table 8:** Proportions of the type series of *Brachycephalus verrucosus*.

	$\bar {X}$	SD	Range
THL/SVL	35%	1%	33–38%
TBL/THL	90%	7%	70–100%
HL/SVL	33%	2%	30–36%
ED/HL	34%	3%	30–40%
HW/HL	118%	10%	110–138%

**Notes.**

$\bar {X}$ mean SD standard deviation

Character abbreviations listed in the Material and Methods.

### Etymology

The specific epithet is from the Latin verrucosus (“warty,” “rugged”) in reference to the highly rugose aspect of the dorsum of the body of the new species.

### Distribution

*Brachycephalus verrucosus* is known only from the type locality.

### Remarks

Individuals of the new species were found on leaf litter at montane forest (“Floresta Ombrófila Densa Montana”) between 455–945 m a.s.l. During visits to the type locality, we found individuals of *B. verrucosus* hidden in the leaf litter, some of which vocalizing.

**Table:** 

**Figure 9:**
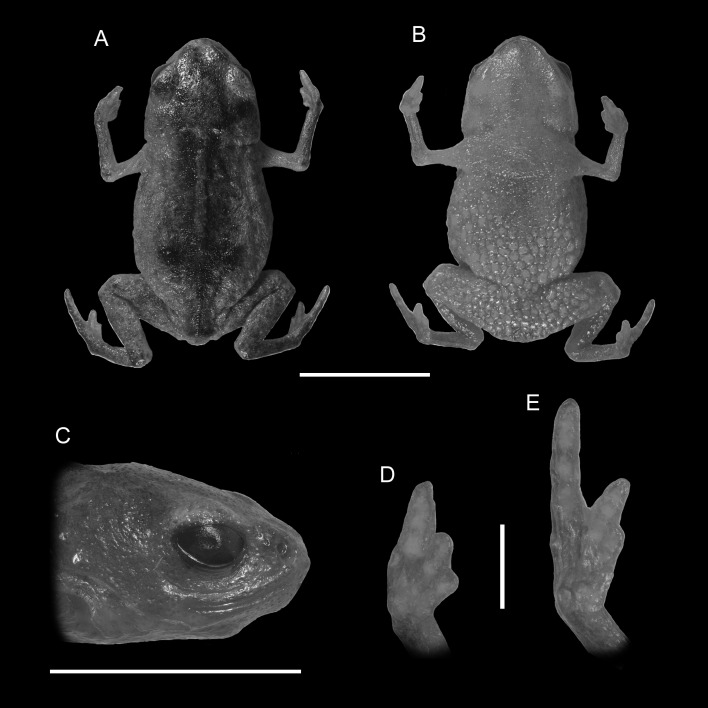
Holotype of *Brachycephalus fuscolineatus* (DZUP 159). (A) Dorsal view of the body, (B) ventral view of the body, (C) lateral view of the head, (D) ventral view of right hand, (E) ventral view of right foot. Horizontal scale bar = 5 mm; vertical scale bars = 2 mm.

**Figure 10:**
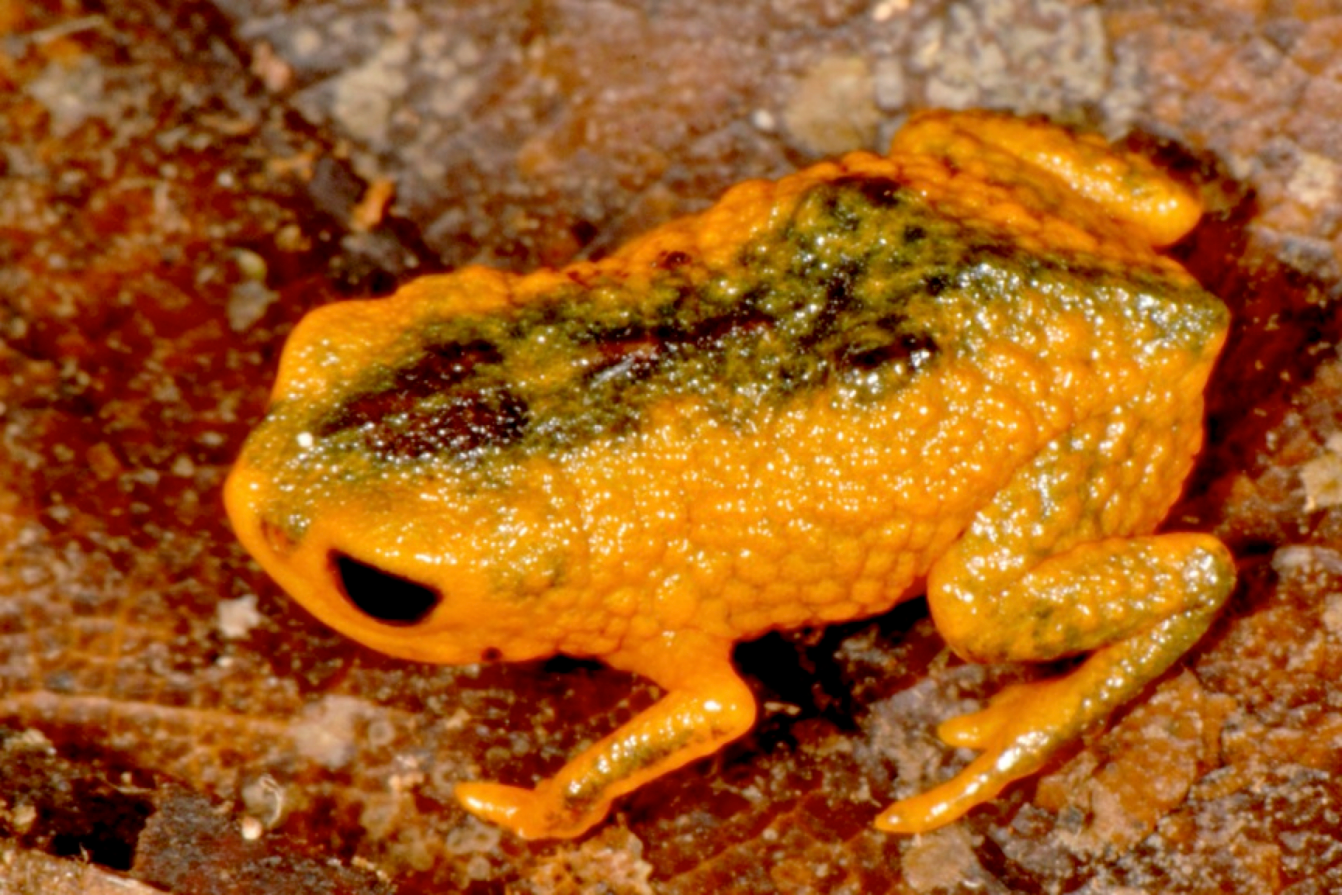
*Brachycephalus fuscolineatus* in life.

*Brachycephalus fuscolineatus* sp. nov.
Pie, Bornschein, Firkowski, Belmonte-Lopes & Ribeiro
(Figs. 9 and 10)
*Brachycephalus* sp. nov. 9 (Pie et al., 2013)
Urn:lsid:zoobank.org:act: 037B088C-AAA2-4BD4-B6E9-DFFF7B753D8E

### Holotype

DZUP 159 (Fig. 9), adult male collected at Morro do Baú (26°47′58″S, 48°55′47″W; 680 m a.s.l.), municipality of Ilhota, state of Santa Catarina, southern Brazil, by MRB and RB-L on 23 October 2010.

### Paratopotypes

DZUP 158 (juvenile), 160, collected with the holotype by MRB and RB-L; and DZUP 401–5, collected by MRP, LFR and Felipe Cini on 28 February 2012, between 640–790 m a.s.l.

### Diagnosis

*Brachycephalus fuscolineatus* is distinguished from all of the species in the genus by the following combination of characters: Body robust, bufoniform, adult SVL 9.7–12.4 mm, rough dorsum (Fig. 9); general coloration predominantly yellow, with a stripe along the vertebral column varying from dark brown to black; skin on dorsum of head and central body dorsum with no dermal co-ossification (Fig. 10). A representative of the *pernix* group, its rugose body dorsum is similar to that of *B. mariaeterezae*, *B. olivaceus*, *B. auroguttatus* and *B. verrucosus*, as opposed to the smooth dorsum of *B. izecksohni*, *B. brunneus*, *B. pernix*, *B. ferruginus*, *B. pombali*, and *B. tridactylus*). The brown to black stripe along the dorsum of *B. fuscolineatus* is similar to *B. ferruginus*, although the orange coloration on the sides and belly of the new species differs from the yellow coloration on the sides and belly of *B. ferruginus*. The new species lacks the dermal co-ossification characteristic of species of the *ephippium* group, and the bufoniform shape and larger body size of the new species distinguish it from those in the *didactylus* group, which are smaller (SVL = 8–10 mm) and have leptodactyliform body shape.

### Description of holotype

Body robust, bufoniform; head slightly wider than long; head length 30% of snout-vent length; snout short, with length almost equal to eye diameter, rounded in dorsal and lateral views; nostrils not protuberant, directed anterolaterally; canthus rostralis indistinct; lips nearly sigmoid; loreal region slightly concave; eye slightly protuberant in dorsal and lateral views; ED 40% of head length; tympanum indistinct; vocal sac not expanded externally; vocal slits present; tongue longer than wide, with posterior half not adherent to floor of mouth; choanae relatively small and round; vomerine odonthophores absent; arm and forearm relatively slender; arm approximately as long as forearm; tip of finger I and II slightly rounded, tip of finger III pointed; finger I and IV very small, vestigial; relative lengths of fingers IV < I < II < II; subarticular tubercles absent; inner and outer metacarpal tubercles absent; legs short, moderately robust; thigh length 36% of snout-vent length, tibia length 86% of thigh length; toes II–III short, relatively distinct; toes I and V externally absent; relative length of toes II < III < IV; subarticular tubercles and inner metatarsal tubercles absent; outer metatarsal tubercle distinct, large, ovoid; dorsum granular, without co-ossifications; glandular warts large, circular, juxtaposed on dorsum and legs; numerous small glandular warts near the vertebral column and on head and arms; sides of the body granular; large glandular warts circular and juxtaposed on the sides of the body; belly granular; circular glandular warts juxtaposed, distributed from belly to legs; chin and arms smooth.

### Coloration of holotype

In life, a dark-brown to black stripe on the central region of the dorsum of the head and extending along the anteroposterior region of the dorsum of the body. Irregular black spots becoming increasingly infrequent along the dorsal region of arms, thighs, and legs; peripheral region of the dorsum, sides of the body, arms, legs, thighs, belly, chin, ventral region of the hands and feet orange; spots around nostrils black and irregular; iris black (Fig. 10). In preservative, the dark stripe along the dorsum maintains the same color. The orange regions of the dorsum and sides become light gray, whereas the remaining originally orange regions become pale cream.

### Measurements of holotype

SVL = 11.0 mm, HL = 3.3 mm, HW = 4.1 mm, ED = 1.3 mm, ND = 0.2 mm, IOD = 2.3 mm, IND = 1.3 mm, END = 0.7 mm, THL = 4.0 mm, TBL = 3.4 mm.

### Variation in type series

Measurements and proportions are given in Tables 9 and 10. The diagnostic dark stripe on their back varies both in width and in the sinuosity of their edges.

**Table 9:** Measurements (mm) of the type series of *Brachycephalus fuscolineatus*.

Trait	$\bar {X}$	SD	Range
SVL	10.97	0.91	9.7–12.4
HL	3.41	0.24	3.1–3.9
HW	4.11	0.16	3.9–4.3
ED	1.34	0.10	1.2–1.5
ND	0.19	0.02	0.2–0.2
IOD	2.30	0.12	2.2–2.6
IND	1.26	0.09	1.2–1.4
END	0.66	0.04	0.6–0.7
THL	4.09	0.30	3.9–4.7
TBL	3.66	0.30	3.4–4.1

**Notes.**

$\bar {X}$ mean SD standard deviation

*N* = 7 specimens.

Character abbreviations listed in the Material and Methods.

**Table 10:** Proportions of the type series of *Brachycephalus fuscolineatus*.

	$\bar {X}$	SD	Range
THL/SVL	37%	2%	33–40%
TBL/THL	90%	4%	86–100%
HL/SVL	31%	1%	29–34%
ED/HL	39%	1%	36–41%
HW/HL	121%	5%	111–126%

**Notes.**

$\bar {X}$ mean SD standard deviation

Character abbreviations listed in the Material and Methods.

### Etymology

The specific epithet is from the Latin fuscus (“dark,” “swarthy,” “dark-skinned”) and lineatus (“of a line”), in reference to the characteristic dark stripe across the dorsum of the new species.

### Distribution

*Brachycephalus fuscolineatus* is known only from the type locality.

### Remarks

Individuals of the new species were found on leaf litter at montane forest (“Floresta Ombrófila Densa Montana”) between 640–790 m a.s.l. During visits to the type locality, we found specimens of *B. fuscolineatus* hidden in the leaf litter, some of which vocalizing.

**Table:** 

**Figure 11:**
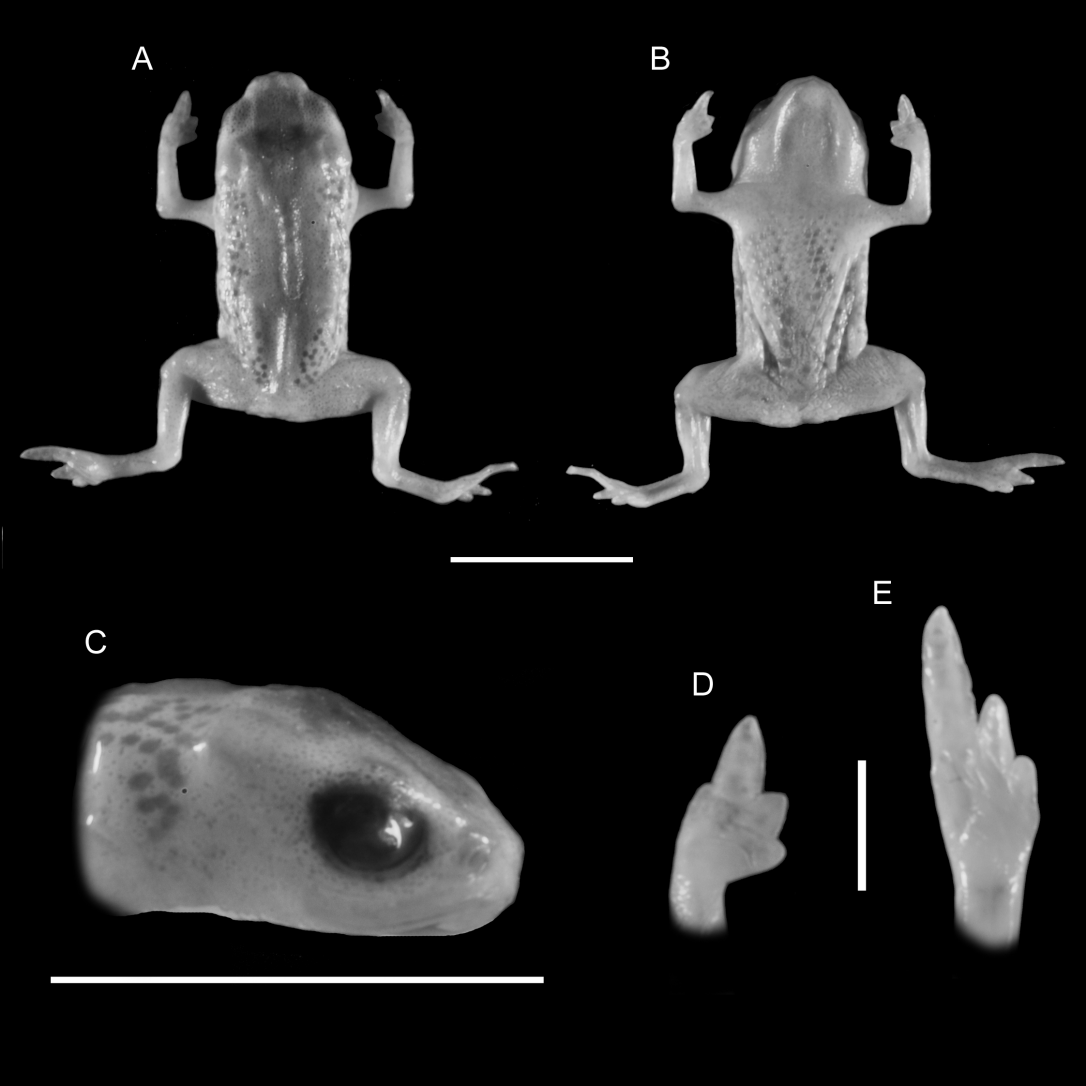
Holotype of *Brachycephalus leopardus* (DZUP 490). (A) Dorsal view of the body, (B) ventral view of the body, (C) lateral view of the head, (D) ventral view of right hand, (E) ventral view of right foot. Horizontal scale bar = 5 mm; vertical scale bars = 2 mm.

**Figure 12:**
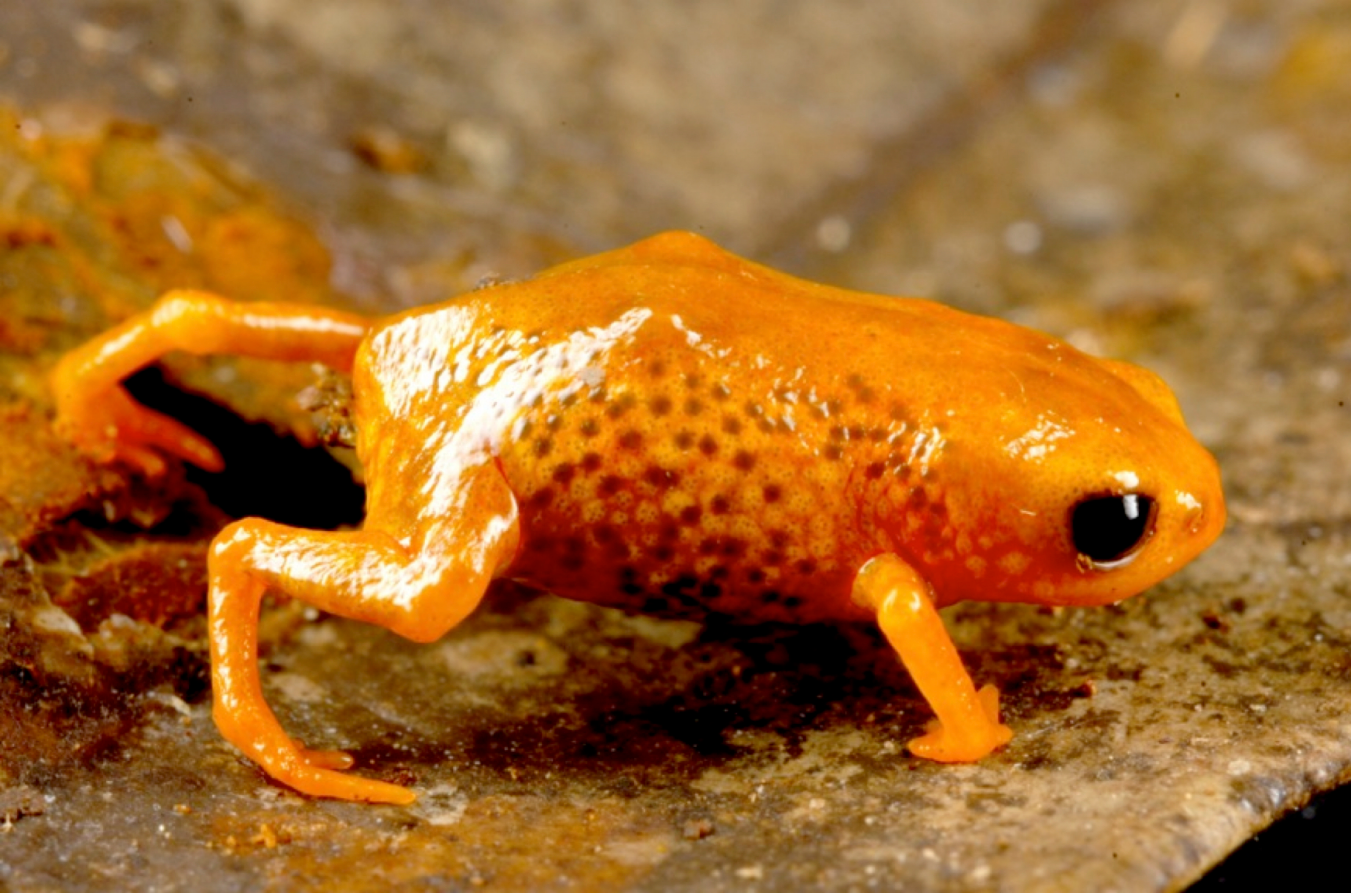
*Brachycephalus leopardus* in life.

**Figure 13:**
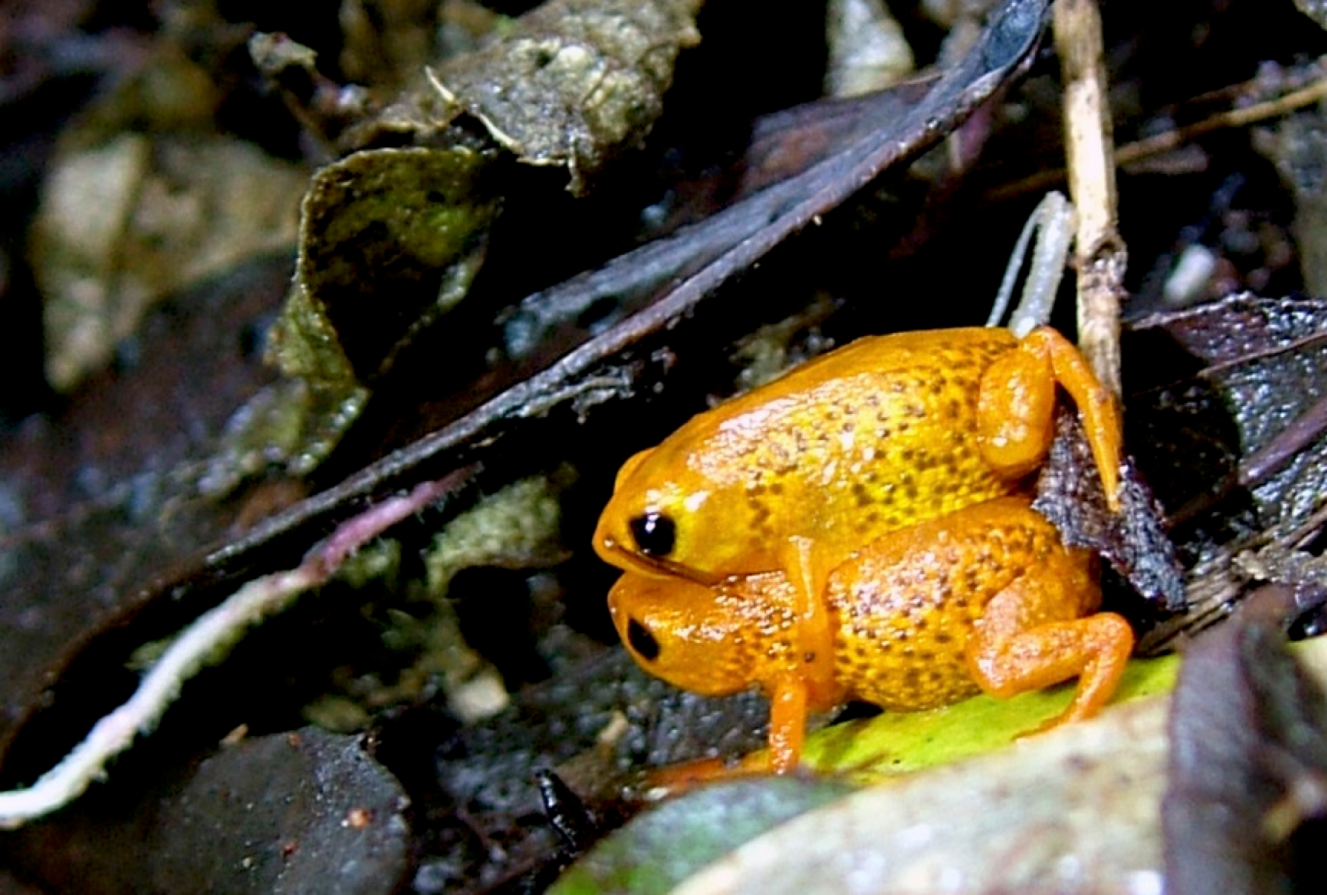
*Brachycephalus leopardus* during amplexus.

*Brachycephalus leopardus* sp. nov.
Ribeiro, Firkowski & Pie
(Figs. 11–13)
*Brachycephalus* sp. nov. 4 (Pie et al., 2013)
Urn:lsid:zoobank.org:act: 257833FA-F201-4FB8-873C-EDF740621804

### Holotype

DZUP 490 (Fig. 11), adult male collected at Serra do Araçatuba (25°54′07″S, 48°59′47″W; 1,640 m a.s.l.), municipality of Tijucas do Sul, state of Paraná, southern Brazil, on 06 January 2006 by Marcelo Leandro Brotto and LFR.

### Paratopotypes

DZUP 478–89, 491, 492 (juvenile), 13 adults collected with the holotype by Marcelo Leandro Brotto and LFR.

### Diagnosis

*Brachycephalus leopardus* is distinguished from all of the species in the genus by the following combination of characters: Body robust, bufoniform, adult SVL 9.7–11.9 mm. smooth dorsum (Fig. 11); coloration orange on along the vertebral column, varying to yellow along the body flanks, which become increasingly verrucose; skin on dorsum of head and central body dorsum with no dermal co-ossification (Fig. 12). The smooth dorsum of *B. leopardus* is similar to that of *B. izecksohni*, *B. brunneus*, *B. pernix*, *B. ferruginus*, *B. pombali*, and *B. tridactylus* (as opposed to the rugose dorsum of *B. mariaeterezae*, *B. olivaceus*, *B. auroguttatus*, *B. verrucosus*, and *B. fuscolineatus*). The new species is unique among all *Brachycephalus* species in the presence of minute dark spots on the dorsal portion of the head, thorax, legs, and arms, while also displaying larger dark spots on the sides of the body. The new species lacks the dermal co-ossification characteristic of species of the *ephippium* group, and the bufoniform shape and larger body size of the new species distinguish it from those in the *didactylus* group, which are smaller (SVL = 8–10 mm) and have leptodactyliform body shape.

### Description of holotype

Body robust, bufoniform (Fig. 11); head slightly wider than long (Figs. 11A and 11B); head length 32% of snout-vent length; snout short, with length almost equal to eye diameter, slightly truncate in dorsal and lateral views (Figs. 11A and 11B) nostrils protuberant, directed anterolaterally (Figs. 11A and 11B); canthus rostralis indistinct; lips nearly sigmoid; loreal region slightly concave; eye slightly protuberant in dorsal and lateral views; ED 36% of head length; tympanum indistinct; vocal sac not expanded externally; vocal slits present; tongue longer than wide, with posterior half not adherent to floor of mouth; choanae relatively small and round; vomerine odonthophores absent; arm and forearm relatively slender; arm approximately as long as forearm; tip of finger I slightly rounded, tip of fingers II and III pointed; finger I and IV very small, vestigial; relative lengths of fingers IV < I < II < III; subarticular tubercles absent; inner and outer metacarpal tubercles absent; legs short, moderately robust; thigh length 35% of snout-vent length, tibia length 84% of thigh length; toes II–III short, relatively distinct; toe I externally absent, toe V very reduced, vestigial; relative length of toes V < II < III < IV; subarticular tubercles and inner metatarsal tubercles absent; outer metatarsal tubercle distinct, large, ovoid; dorsum smooth, without co-ossifications; arms, legs, and head smooth; sides of the body granular; large glandular warts circular and not juxtaposed on the sides of the body; belly granular; large glandular warts circular, not juxtaposed on belly and thighs; chin, arms, and legs smooth.

### Coloration of holotype

In life, overall orange coloration shifting to yellow on the sides of the body and belly; dorsum, belly, arms, legs, thighs, hands, and feet covered with minute dark spots; larger dark spots scattered along the sides of the body; iris black (Fig. 12). In preservative, general color cream, with sides of the body pale cream, with both large and small spots still remaining apparent.

### Measurements of holotype

SVL = 10.3 mm, HL = 3.3 mm, HW = 3.9 mm, ED = 1.3 mm, ND = 0.2 mm, IOD = 2.2 mm, IND = 1.2 mm, END = 0.6 mm, THL = 3.6 mm, TBL = 3.0 mm.

### Variation in type series

Measurements and proportions are given in Tables 11 and 12. The extent of the yellow region on the sides of the body and belly might vary among individuals, as well as the density of both large and small spots on the sides and in the dorsal region.

**Table 11:** Measurements (mm) of the type series of *Brachycephalus leopardus*.

Trait	$\bar {X}$	SD	Range
SVL	10.82	0.62	9.7–11.9
HL	3.55	0.27	3.1–4.0
HW	3.99	0.19	3.7–4.3
ED	1.33	0.09	1.2–1.5
ND	0.18	0.02	0.2–0.2
IOD	2.27	0.11	2.1–2.5
IND	1.25	0.08	1.1–1.4
END	0.62	0.05	0.6–0.7
THL	3.67	0.31	3.1–4.1
TBL	3.20	0.18	3.0–3.4

**Notes.**

$\bar {X}$ mean SD standard deviation

*N* = 14 specimens.

Character abbreviations listed in the Material and Methods.

**Table 12:** Proportions of the type series of *Brachycephalus leopardus*.

	$\bar {X}$	SD	Range
THL/SVL	34%	2%	29–38%
TBL/THL	87%	5%	81–100%
HL/SVL	33%	1%	31–35%
ED/HL	38%	3%	34–43%
HW/HL	113%	5%	107–123%

**Notes.**

$\bar {X}$ mean SD standard deviation

Character abbreviations listed in the Material and Methods.

### Etymology

The specific epithet is from the Latin leopardus and refers to the spotted pattern reminiscent of the coloration of the felid genus *Leopardus*.

### Distribution

*Brachycephalus leopardus* is known from the type locality and from the Morro dos Perdidos (25°53′22″S, 48°57′22″W; 1,410 m a.s.l.), municipality of Guaratuba, state of Paraná (Pie et al., 2013).

### Remarks

Individuals were found on leaf litter in patches of cloud forest (“Floresta Ombrófila Densa Altomontana). Individuals were active by day, and calling adult males were never found calling exposed on top of leaf litter. Axillary amplexus was recorded in the species (Fig. 13).

**Table:** 

**Figure 14:**
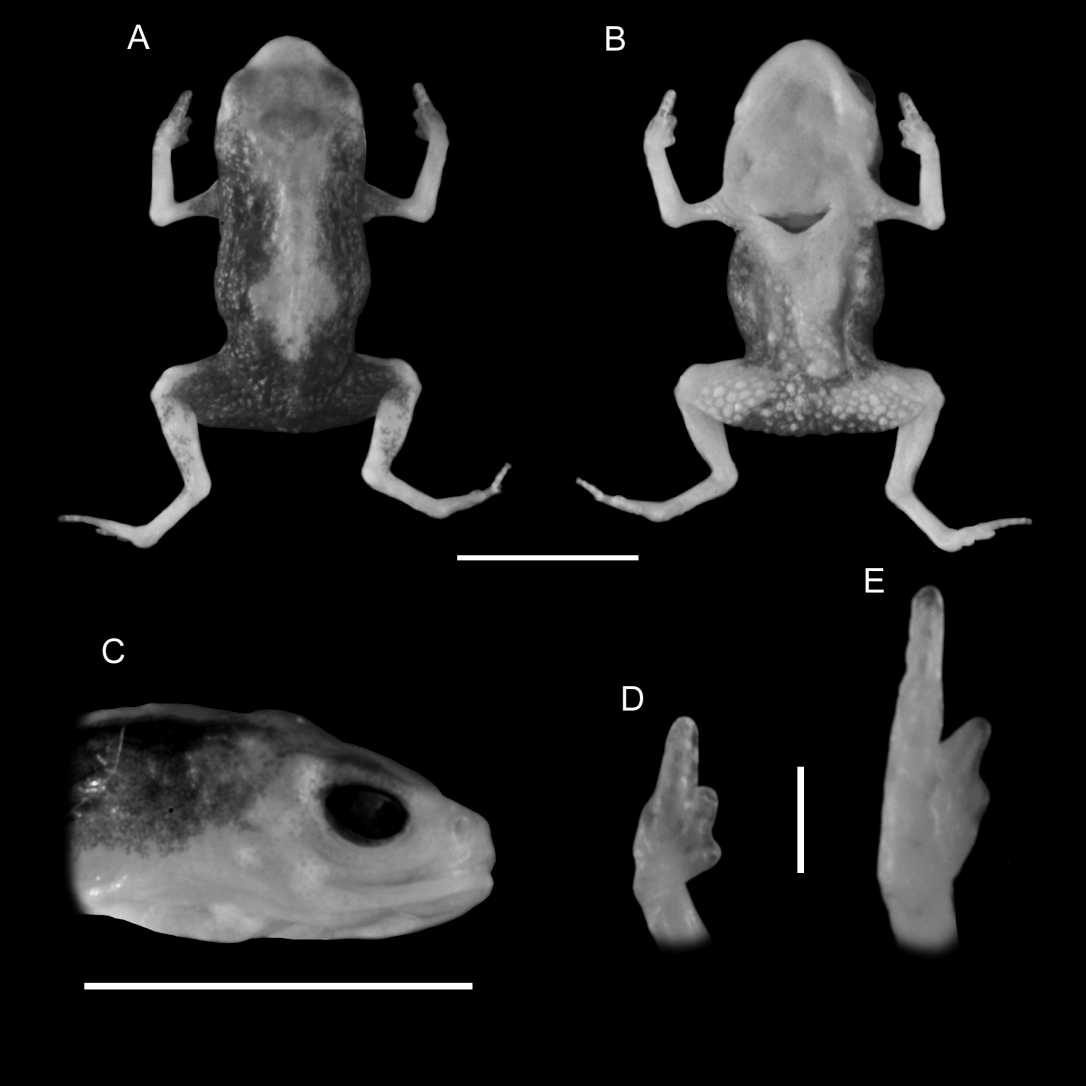
Holotype of *Brachycephalus boticario* (DZUP 440). (A) Dorsal view of the body, (B) ventral view of the body, (C) lateral view of the head, (D) ventral view of right hand, (E) ventral view of right foot. Horizontal scale bar = 5 mm; vertical scale bars = 2 mm.

**Figure 15:**
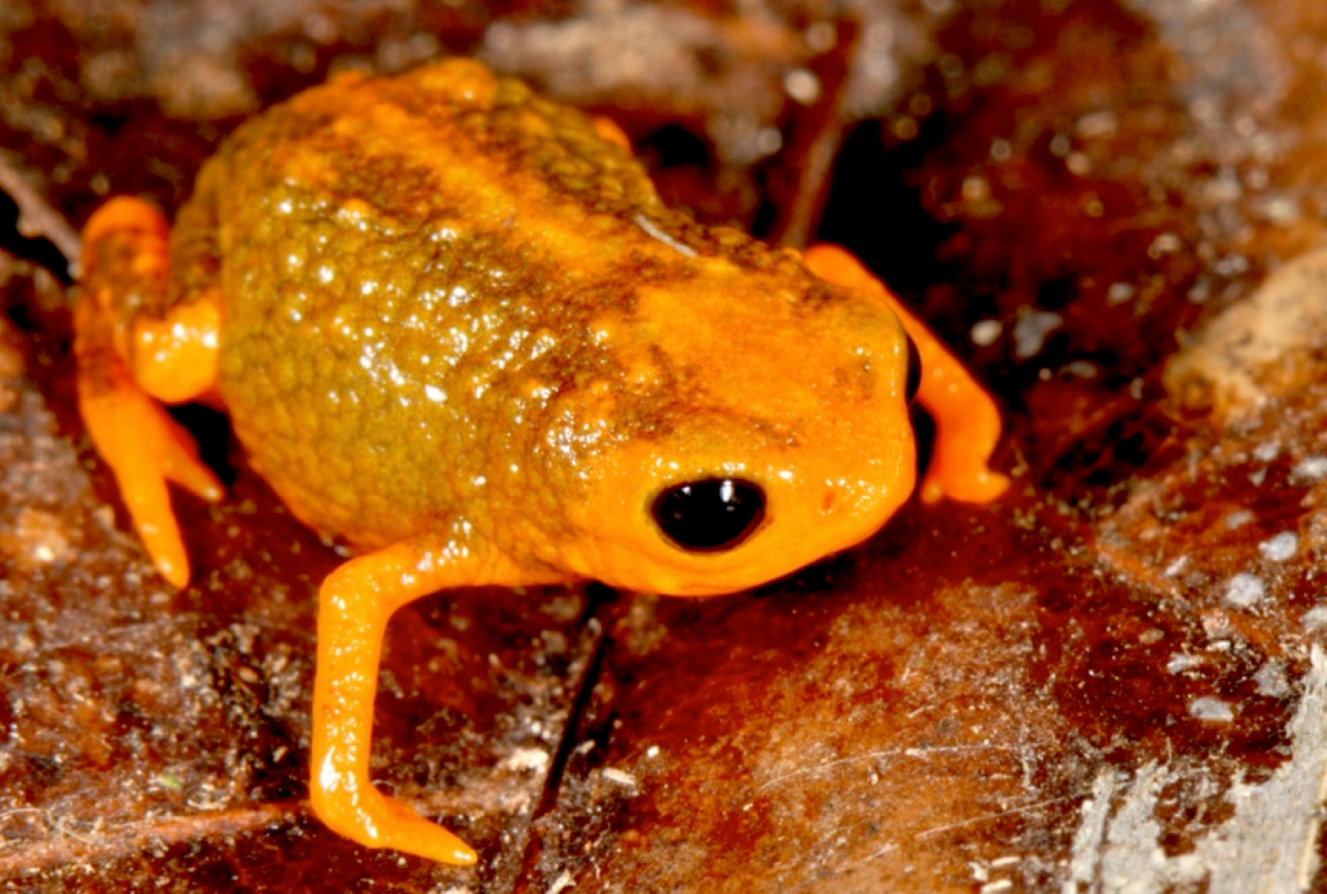
*Brachycephalus boticario* in life.

*Brachycephalus boticario* sp. nov.
Pie, Bornschein, Firkowski, Belmonte-Lopes & Ribeiro
(Figs. 14 and 15)
Urn:lsid:zoobank.org:act: 13308B44-84CB-4A99-88CC-FC0A83EBFFE9

### Holotype

DZUP 440 (Fig. 14), adult male collected at Morro do Cachorro (26°46′42″S, 49°01′57″W; 795 m a.s.l.), on the border between the municipalities of Blumenau, Gaspar, and Luiz Alves, state of Santa Catarina, southern Brazil, on 29 October 2012 by MRP and LFR.

### Paratopotypes

DZUP 414-5, 438-9, 444-5, six adult collected with the holotype by the same researchers, and DZUP 459, one adult collected on 23 January 2013 by MRP, Felipe Cini, and LFR, between 755–795 m a.s.l.

### Diagnosis

*Brachycephalus boticario* is distinguished from all of the species in the genus by the following combination of characters: Body robust, bufoniform, adult SVL 10.0–12.7 mm, rough dorsum (Fig. 14); overall coloration light-brown, becoming yellow on the ventral region of the legs and arms, on the dorsum of the head and as a stripe along the vertebral column; skin on dorsum of head and central body dorsum with no dermal co-ossification (Fig. 15). A representative of the *pernix* group with an overall appearance highly similar to *B. pernix*, but distinct from that species by its rugose dorsum (which is smooth in *B. pernix*). The rugose body dorsum is also similar to that of *B. mariaeterezae*, *B. olivaceus*, *B. auroguttatus*, *B. verrucosus*, and *B. fuscolineatus*, as opposed to the smooth dorsum of *B. izecksohni*, *B. brunneus*, *B. ferruginus*, *B. pombali*, *B. tridactylus*, and *B. leopardus*. The new species lacks the dermal co-ossification characteristic of species of the *ephippium* group, and the bufoniform shape and larger body size of the new species distinguish it from those in the *didactylus* group, which are smaller (SVL = 8–10 mm) and have leptodactyliform body shape.

### Description of holotype

Body robust, bufoniform; head slightly wider than long; head length 34% of snout-vent length; snout short, with length almost equal to eye diameter, rounded in dorsal and lateral views; nostrils protuberant, directed anterolaterally; canthus rostralis faintly distinct; lips nearly sigmoid; loreal region slightly concave; eye slightly protuberant in dorsal and lateral views; ED 31% of head length; tympanum indistinct; vocal sac not expanded externally; vocal slits present; tongue longer than wide, with posterior half not adherent to floor of mouth; choanae relatively small and round; vomerine odonthophores absent; arm and forearm relatively slender; arm approximately as long as forearm; tip of finger I and II slightly rounded, tip of finger III pointed; finger I and IV very small, vestigial; relative lengths of fingers IV < I < II < II; subarticular tubercles absent; inner and outer metacarpal tubercles absent; legs short, moderately robust; thigh length 33% of snout-vent length, tibia length 96% of thigh length; toes II–III short, relatively distinct; toe I very reduced, toe V externally absent; relative length of toes II < III < IV; subarticular tubercles and inner metatarsal tubercles absent; outer metatarsal tubercle distinct, large, ovoid; dorsum granular, without co-ossifications; head, arms and legs smooth; glandular warts circular, almost juxtaposed; sides of the body granular, with glandular warts circular, almost juxtaposed; belly granular; glandular warts circular in belly, thighs, legs, and arms; chin smooth.

### Coloration of holotype

In life, dorsum orange from the head to the pelvic region; posterior dorsal and cloacal regions light brown; arms and legs orange, at times with irrregular light-brown spots on the dorsal surface; head with spots irregular and light-brown near the eyes; sides of the body light brown from the sides of the head to the middle of the body; belly, chin, arms, and legs, in ventral view, orange; ventral surface of the thighs with irregular light-brown spots near the inguinal region; iris black (Fig. 15). In preservative, the originally orange regions become light gray in the dorsum and pale cream elsewhere, whereas the brown regions retain their original color.

### Measurements of holotype

SVL = 10.9 mm, HL = 3.7 mm, HW = 4.0 mm, ED = 1.2 mm, ND = 0.2 mm, IOD = 2.2 mm, IND = 1.2 mm, END = 0.6 mm, THL = 3.6 mm, TBL = 3.4 mm.

### Variation in type series

Measurements and proportions are given in Tables 13 and 14. The extent of the orange coloration of the dorsum might vary from a thin, irregular line to a thicker stripe across their dorsum.

**Table 13:** Measurements (mm) of the type series of *Brachycephalus boticario*.

Trait	$\bar {X}$	SD	Range
SLV	11.06	0.85	10.0–12.7
HL	3.70	0.18	3.6–4.0
HW	4.24	0.24	3.9–4.6
ED	1.19	0.05	1.2–1.3
ND	0.13	0.05	0.1–0.2
IOD	2.19	0.10	2.0–2.4
IND	1.22	0.08	1.1–1.4
END	0.63	0.06	0.6–0.8
THL	3.81	0.17	3.6–4.0
TBL	3.47	0.23	3.3–4.0

**Notes.**

$\bar {X}$ mean SD standard deviation

*N* = 8 specimens.

Character abbreviations listed in the Material and Methods.

**Table 14:** Proportions of the type series of *Brachycephalus boticario*.

	$\bar {X}$	SD	Range
THL/SVL	34%	3%	30–39%
TBL/THL	93%	6%	86–104%
HL/SVL	34%	2%	31–36%
ED/HL	32%	1%	30–34%
HW/HL	113%	4%	108–119%

**Notes.**

$\bar {X}$ mean SD standard deviation

Character abbreviations listed in the Material and Methods.

### Etymology

The specific epithet is a homage to the Fundação Grupo Boticário de Proteção à Natureza, which not only partially funded the fieldwork of this study, but also is a major contributor to conservation research in Brazil.

### Distribution

*Brachycephalus boticario* is known only from the type locality.

### Remarks

Individuals of the new species were found on leaf litter of a cloud forest (“Floresta Ombrófila Densa Montana”) at 755–795 m a.s.l. During visits to the type locality, we found individuals hidden in the leaf litter, some of which vocalizing.

## Discussion

**Figure 16:**
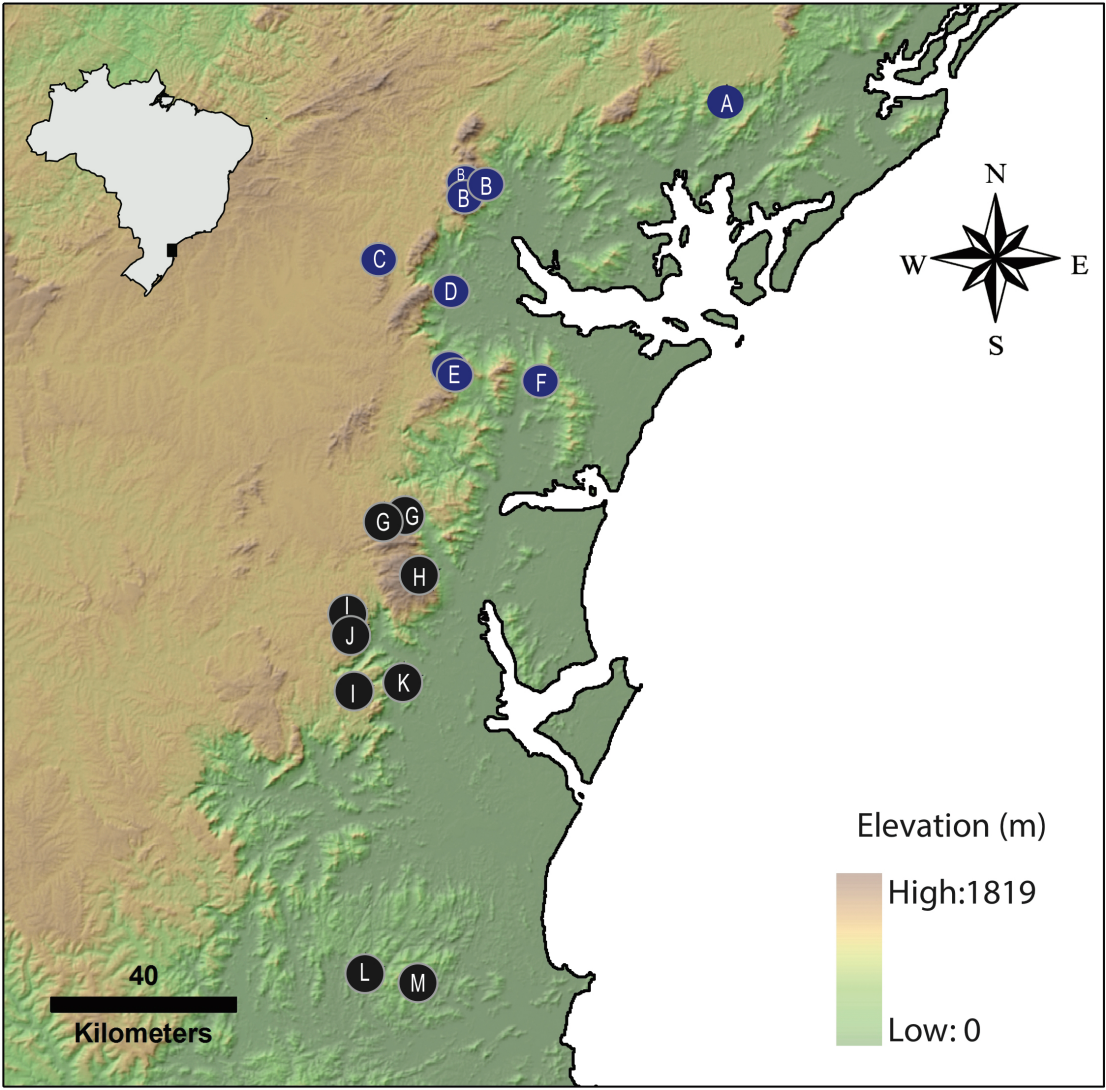
Geographical distribution of *Brachycephalus* species of the pernix group. Blue symbols represent occurrence records of previously described species, based on Pie et al. (2013), whereas black symbols indicate the new species described in the present study. Previously described species: A, *B. tridactylus*; B, *B. brunneus*; C, *B. pernix*; D, *B. ferruginus*; E, *B. pombali*; F, *B. izecksohni*. Species described herein: G, *B. leopardus*; H, *B. auroguttatus*; I, *B. olivaceus*; J, *B.mariaeterezae*; K, *B. verrucosus*; L, *B. boticario*; M, *B. fuscolineatus*. The black triangle on the inset with the map of Brazil indicates the position of the area in the larger map.

In this study we more than double the number of species in the *pernix* group, which now formally extends into the state of Santa Catarina (Fig. 16). Such high success in uncovering new species might indicate that the total number of *Brachycephalus* is still underestimated. Interestingly, during field expeditions over the course of this study, we failed to find *Brachycephalus* in a private reserve (RPPN Prima Luna) in the municipality of Nova Trento, Santa Catarina, which seemed to provide conditions for the presence of *Brachycephalus* such as the presence of cloud forest, well-preserved leaf litter and high altitude. This absence could suggest that the southern limit of the genus coincides with the Itajaí valley, in the state of Santa Catarina.

It is important to note that, although there is slight intraspecific morphological variation in every new species described in the present study, such variation is small compared to the substantial differences among species (Table 15). Moreover, the observed variation is comparable to that found among most currently recognized *Brachycephalus* species, thus rejecting the possibility that we were dealing with a single, highly variable species. In addition, genetic data using phylogenomic data (>800 nuclear loci) were consistent with the species delimitations used in the present study (Pie et al., 2015, unpublished data), with extensive analyses being prepared for publication in a later study. The availability of genetic data, as well as the study of the natural history of these new species, such as diet and vocalizations, should provide valuable insight into their evolutionary history and speciation.

**Table 15:** Main diagnostic traits of species of the pernix group.

Species	Body size (SVL in mm)	Coloration of dorsum	Coloration of venter	Snout shape	Presence of toe V	Shape of outer metacarpal tubercle	Skin texture on dorsum
*B. izecksohni*	10.3–13.1	Orange with lateral small glands dark orange with small olive grey central points on the sides of the body	Orange	Rounded in dorsal and lateral views	Absent	Distinct, large, ovoid	Smooth
*B. brunneus*	9.3–12.0	Dark brown	Varying dark brown with irregular orange spots to nearly completely orange	Mucronate in dorsal view, rounded in lateral view	Very reduced	Distinct, large, ovoid	Smooth
*B. ferruginus*	11.6–14.5	Orange with dorsal reddish–brown irregular markings, lateral surfaces with small dark brown spots	Orange with small greenish dots	Rounded in dorsal and lateral views	Absent	Absent	Smooth
*B. pombali*	12.6–15.3	Orange, lateral surfaces with small faint brown spots	Orange with brown spots	Rounded in dorsal and lateral views	Very reduced	Absent	Smooth
*B. pernix*	12.0–15.8	Orange, lateral surfaces with small dark brown spots	Orange	Semicircular in dorsal view, rounded in lateral view	Absent	Absent	Smooth
*B. tridactylus*	10.6–13.82	Orange, lateral surfaces with regular small olive–gray spots	Pale orange to dark orange	Rounded in dorsal and lateral views	Absent	Absent	Smooth
*B. mariaeterezae*	10.4–13.4	Yellow with a light-blue stripe along the vertebral column. Lateral surfaces with small brown spots	Yellow, with small brown spots	Rounded in dorsal and lateral views	Absent	Absent	Rough
*B. olivaceus*	9.4–12.9	Dark-green to brown	Dark-green to brown, with small orange regions in some individuals	Rounded in dorsal and lateral views	Absent	Absent	Rough
*B. auroguttatus*	9.0–13.6	Shifting from bright yellow on the head with increasingly more brown toward the posterior region of the body, with yellow spots along the back	Shifting from bright yellow on the chin with increasingly more brown toward the posterior region of the body	Rounded in dorsal and lateral views	Absent	Absent	Rough
*B. verrucosus*	9.6–13.2	Light-green, with a thin orange stripe along most of the vertebral column in some individuals	Yellow or yellow in the center surrounded by light green	Rounded in dorsal and lateral views	Absent	Absent	Rough
*B. fuscolineatus*	9.7–12.4	Predominantly yellow with a stripe along the vertebral column varying from dark brown to black	Orange	Rounded in dorsal and lateral views	Absent	Absent	Rough
*B. leopardus*	9.7–11.9	Orange, shifting to yellow on the sides of the body or yellow; sides of the body with large dark spots.	Orange or yellow covered with dark spots; large dark spots scattered along the venter	Rounded in dorsal and lateral views	Very reduced	Absent	Smooth
*B. boticario*	10.0–12.7	Light-brown with orange stripe along the vertebral column and yellow sides	Orange	Rounded in dorsal and lateral views	Absent	Absent	Rough

Although little is known about the natural history of the new species described in the present study, assessments over the course of our fieldwork raise concern about their conservation status. Cloud forests in southern Brazil have experienced disturbances from a variety of sources, particularly from (often illegal) deforestation for pine tree plantations and extensive cattle ranching. Although the latter might involve relatively low densities of cattle, their foraging activity leads to severe trampling of the leaf litter, which provides the delicate combination of conditions that allow for the persistence of *Brachycephalus* populations. More incisive actions by state environmental agencies (Instituto Ambiental do Paraná in the state of Paraná and Fundação do Meio Ambiente in the state of Santa Catarina) to manage these activities could be decisive for their long-term conservation. In addition, the species described here should be integrated into the “Plano de Ação Nacional para a Conservação dos Anfíbios e Répteis Ameaçados da Região Sul do Brasil,” an initiative organized by the Instituto Chico Mendes/Ministry of the Environment that seeks to organize conservation initiatives for the conservation of the herpetofauna of southern Brazil.

## Supplemental Information

10.7717/peerj.1011/supp-1 Appendix S1 Examined specimens

10.7717/peerj.1011/supp-2 Figure S1 Variation in dorsal coloration of *Brachycephalus mariaeterezae* (DZUP 393-9) Specimens had been sacrificed minutes prior to the photograph. The dark coloration on the dorsum of the head is a post-mortem effect that is not present in live specimens. Scale bar = 1 cm.

## Appendix. Examined specimens

*Brachycephalus brunneus*. PARANÁ: Caratuva, Serra dos Órgãos, municipality of Campina Grande do Sul MHNCI 1919-20, MNRJ 40,289-91 (paratypes).

*Brachycephalus didactylus*. RIO DE JANEIRO: municipality of Engenheiro Paulo de Frontin ZUEC 1133, 10825, 1132, MZUSP 94621; Sacra Família do Tinguá, municipality of Engenheiro Paulo de Frontin MZUSP 13613-20, 64810-1.

*Brachycephalus ephippium*. RIO DE JANEIRO: Parque Nacional Serra dos Órgãos, MZUSP 104140-7.

*Brachycephalus ferruginus*. PARANÁ: Olimpo (25°27′03″S, 48°54′59″W), Serra do Marumbi, municipality of Morretes MHNCI 125, 128.

*Brachycephalus hermogenesi*. SÃO PAULO: Ubatuba ZUEC 9715-21, 9723-5. Reserva Florestal de Morro Grande, municipality of Cotia MZUSP 132257-63.

*Brachycephalus izecksohni*. PARANÁ: Torre da Prata, Serra da Prata, boundary of the municipalities of Morretes, Paranaguá, and Guaratuba CFBH 7381-2, 7384 (paratypes).

*Brachycephalus nodoterga*. SÃO PAULO: Santana de Parnaíba, MZUSP 147711-6.

*Brachycephalus pernix*. PARANÁ: Anhangava, Serra da Baitaca, municipality of Quatro Barras CFBH 2,597-8 (paratypes), MHNCI 1,818-9 (paratypes) 1820, 3000–4 (paratypes), MNRJ 17349 (holotype), ZUEC 9433-7 (paratypes), DZUP 539-55

*Brachycephalus pombali.* PARANÁ: Morro dos Padres, Pico da Igreja (25°39′S, 48°51′W), municipality of Guaratuba CFBH 8042 (holotype), 8043-53 (paratypes).

*Brachycephalus tridactylus*. PARANÁ: Serra do Morato, Guaraqueçaba, DZUP493-7.

## Additional Information and Declarations

### Field Study Permissions

The following information was supplied relating to field study approvals (i.e., approving body and any reference numbers): Collection permits for this study were issued by the Fundação Municipal do Meio Ambiente (FUNDEMA)/Municipality of Joinville (001/11-GEMAP), Instituto Ambiental do Paraná (permit 355/11), and ICMBIO (SISBIO 10.500, 22470-2).
