# Supplementary material for: Seven new microendemic species of Brachycephalus (Anura: Brachycephalidae) from southern Brazil
Source: PeerJ. 2015 Jun 4;3:e1011. doi: 10.7717/peerj.1011 (PMC4458131; doi:10.7717/peerj.1011)
Supplement: Appendix S1 [file peerj-03-1011-s001.doc]

Appendix I. Examined specimens.

*Brachycephalus brunneus.* PARANÁ: Caratuva, Serra dos Órgãos, municipality of Campina Grande do Sul MHNCI 1919-20, MNRJ 40289-91 (paratypes).

*Brachycephalus didactylus.* RIO DE JANEIRO: municipality of Engenheiro Paulo de Frontin ZUEC 1133, 10825, 1132, MZUSP 94621; Sacra Família do Tinguá, municipality of Engenheiro Paulo de Frontin MZUSP 13613-20, 64810-1.

*Brachycephalus ephippium.* RIO DE JANEIRO: Parque Nacional Serra dos Órgãos, MZUSP 104140-7.

*Brachycephalus ferruginus.* PARANÁ: Olimpo (25°27'03"S, 48°54'59"W), Serra do Marumbi, municipality of Morretes MHNCI 125, 128.

*Brachycephalus hermogenesi.* SÃO PAULO: Ubatuba ZUEC 9715-21, 9723-5. Reserva

Florestal de Morro Grande, municipality of Cotia MZUSP 132257-63.

*Brachycephalus izecksohni.* PARANÁ: Torre da Prata, Serra da Prata, boundary of the

municipalities of Morretes, Paranaguá, and Guaratuba CFBH 7381-2, 7384 (paratypes).

*Brachycephalus nodoterga.* SÃO PAULO: Santana de Parnaíba, MZUSP 147711-6.

*Brachycephalus pernix*. PARANÁ: Anhangava, Serra da Baitaca, municipality of Quatro Barras CFBH 2597-8 (paratypes), MHNCI 1818-9 (paratypes) 1820, 3000-4 (paratypes), MNRJ 17349 (holotype), ZUEC 9433-7 (paratypes), DZUP 539-55

*Brachycephalus pombali.* PARANÁ: Morro dos Padres, Pico da Igreja (25°39'S, 48°51'W), municipality of Guaratuba CFBH 8042 (holotype), 8043-53 (paratypes).

*Brachycephalus tridactylus.* PARANÁ: Serra do Morato, Guaraqueçaba, DZUP493-7.
