# Supplementary figures and images for: Seven new microendemic species of Brachycephalus (Anura: Brachycephalidae) from southern Brazil
Source: PeerJ. 2015 Jun 4;3:e1011. doi: 10.7717/peerj.1011 (PMC4458131; doi:10.7717/peerj.1011)

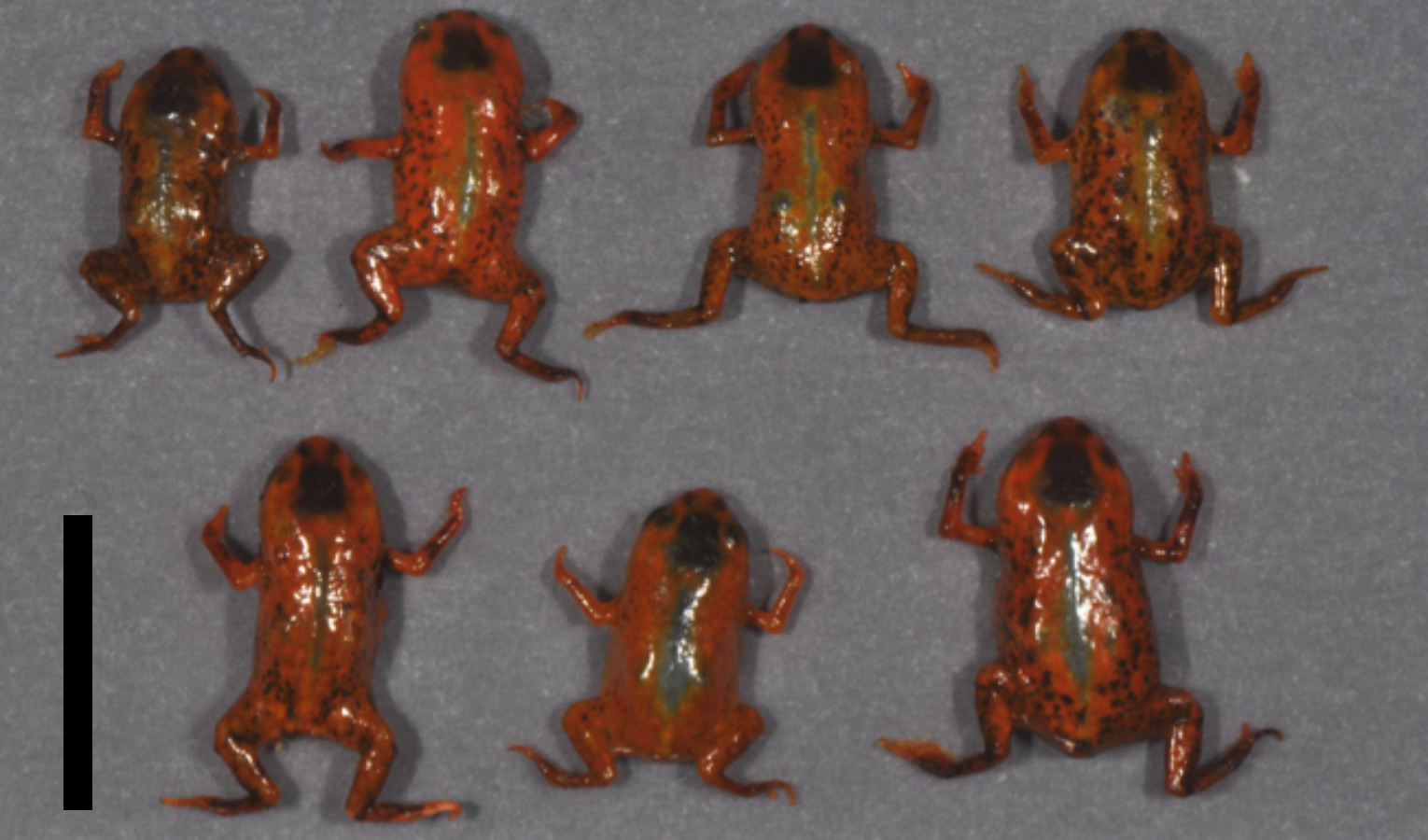

Supplement: Figure S1 — Specimens had been sacrificed minutes prior to the photograph. The dark coloration on the dorsum of the head is a post-mortem effect that is not present in live specimens. Scale bar = 1 cm. [file peerj-03-1011-s002.jpg]
